# The *Drosophila* maternal-effect gene *abnormal oocyte* (*ao*) does not repress histone gene expression

**DOI:** 10.1101/2024.09.17.613536

**Published:** 2024-09-18

**Authors:** Risa Takenaka, Sierra M. Simmerman, Casey A. Schmidt, Eric H. Albanese, Leila E. Rieder, Harmit S. Malik

**Affiliations:** 1 Molecular and Cellular Biology Graduate Program, University of Washington, Seattle WA; 2 Division of Basic Sciences, Fred Hutchinson Cancer Center, Seattle WA 98109; 3 Department of Biology, Emory University, Atlanta GA 30322; 4 Biology Department, Lafayette College, Easton PA 18042; 5 Howard Hughes Medical Institute, Fred Hutchinson Cancer Center, Seattle WA 98109

## Abstract

The *abnormal oocyte* (*ao*) gene of *Drosophila melanogaster* is a maternal-effect lethal gene previously identified as encoding a transcriptional regulator of core histones. However, background genetic mutations in existing *ao* mutant strains could compromise their utility in manipulating histone levels. To distinguish the true *ao* phenotype from background effects, we created two new *ao* reagents: a CRISPR/Cas9-mediated knockout of the *ao* allele for genetic and molecular analyses and an epitope-tagged *ao* allele for cytological experiments. Using these reagents, we confirm previous findings that *ao* exhibits maternal-effect lethality, which can be rescued by either a decrease in the histone gene copy number or by Y chromosome heterochromatin. We also confirm that the Ao protein localizes to the histone locus bodies in ovaries. Our data also suggest that *ao* genetically interacts with the histone genes and heterochromatin, as previously suggested. However, contrary to prior findings, we find that *ao* does not repress core histone transcript levels. Thus, the molecular basis for *ao*-associated maternal-effect lethality remains unknown.

## Introduction

In 1965, Larry Sandler and colleagues collected flies from *Drosophila melanogaster* populations near Rome, Italy, to screen for recessive mutations affecting meiosis. One of the isolated mutants produced excess female offspring when mated to males carrying an attached X^Y chromosome (Sandler et al. 1968; Sandler 1970). Sandler named this mutant *abnormal oocyte* (*abo*, recently renamed *ao*) for its aberrant sex-ratio phenotype (Sandler 1970). Subsequent analyses by Sandler showed that *ao* was one of five maternal-effect, embryonic semi-lethal genes located on the left arm of the 2^nd^ chromosome (Sandler 1977). These five genes shared the unusual property that offspring survival from homozygous-mutant mothers was directly affected by the amount of X and Y heterochromatin in the zygote (Sandler 1977). These genes promised to reveal the mechanistic basis of genetic interactions between euchromatin, heterochromatin, and embryonic viability.

Research on *ao* in the following two decades bolstered Sandler’s initial observation that the viability of offspring from *ao* mutant mothers could be rescued by increasing the dosage of certain heterochromatic regions on the X, Y, and 2^nd^ chromosomes (Parry and Sandler 1974; Sandler 1977; Haemer 1978; Yedvobnick et al. 1980; Pimpinelli et al. 1985; Tomkiel et al. 1991). These regions, located on the distal heterochromatin on the X, the long and short arms of the Y (the *Drosophila* Y chromosome is entirely heterochromatic), and the centromeric heterochromatin on the right arm of the 2^nd^ chromosome, were named AO heterochromatic elements (Pimpinelli et al. 1985).

The mechanistic relationship between the *ao* mutation and AO heterochromatin remains unclear. Sandler hypothesized that an increase in the ribosomal DNA (rDNA) copy number (or the number of rDNA repeats at the locus) was responsible for AO heterochromatin’s amelioration of the maternal-effect lethality. He based this hypothesis on the X- and Y-chromosomal location of the rDNA locus in *D. melanogaster* and the fact that *ao*-associated maternal-effect lethality was lower at 19.5°C (where flies develop slower) than at 25.5°C. Indeed, a subsequent study found that *ao* flies maintained as homozygous mutants developed an expansion of the rDNA locus, which alleviated the maternal-effect lethality (Krider and Levine 1975). Later studies observed the same suppressor phenotype in *ao* flies kept in homozygote stocks. However, while some found additional evidence implicating rDNA (Krider et al. 1979; Graziani et al. 1981), others disputed that rDNA copy number was the cause for the suppressor phenotype (Yedvobnick et al. 1980; Pimpinelli et al. 1985; Sullivan and Pimpinelli 1986; Cavaliere et al. 1991). Furthermore, whether AO heterochromatin rescues the *ao* mutation directly (*i.e*., both *ao* and AO produce the same product) or indirectly (*i.e.*, AO produces a different product than *ao* but performs a rescue function) remained unclear.

Despite over two decades of research, it wasn’t until 1995 that the *ao* mutation was mapped to the cytogenetic locus 32C on the 2^nd^ chromosome of *D. melanogaster* (Tomkiel et al. 1995). The genetic unmasking of *ao* took advantage of two *ao* mutants (*ao*^*1*^, the strain isolated from the Roman fruit-market flies, and *ao*^*2*^, a *P*-element induced allele) and a transgenic rescue construct (Tomkiel et al. 1995). In 2001, the identity of *ao* was revealed as the gene *CG6093* (Berloco et al. 2001), which is the *D. melanogaster* ortholog of the *de-etiolated* or *DET1* gene, first characterized in *Arabidopsis thaliana* but later shown to be present in other plants and animals (Chory et al. 1989; Berloco et al. 2001).

This study also proposed a molecular mechanism underlying *ao*’s maternal-effect lethality (Berloco et al. 2001). It showed that *ao* encoded a protein that localized to the core histone gene promoters. Moreover, it demonstrated that *ao*^*1*^/*ao*^*2*^ trans-heterozygous females produce eggs with significantly increased histone expression levels and that reducing the histone gene copy number in *ao*^*1*^-homozygous females partially ameliorated the *ao*-associated maternal-effect lethality (Berloco et al. 2001). Together, these findings led to the conclusion that the production of excess histones in *ao* mutants caused the maternal-effect lethal phenotype. These results also suggested that heterochromatin could act as a ‘sink’ for excess histones, explaining why excess AO heterochromatin could alleviate the embryonic lethality associated with the loss of maternal *ao*. Thus, this landmark study connected the function of *ao*, a euchromatic gene that controls histone gene expression, with heterochromatin content and, ultimately, embryonic viability.

In *D. melanogaster*, histone genes are arranged in a tandemly repeated, multigene array of approximately 100 units, each comprising all four core histones (H2A, H2B, H3, and H4) and the linker histone (H1) on the 2^nd^ chromosome. Thus, on average, a diploid *D. melanogaster* genome encodes 200 such units, even though histone copy number varies within *D. melanogaster* strains and among *Drosophila* species (Lifton et al. 1978; Strausbaugh and Weinberg 1982; Kremer and Hennig 1990; Mckay et al. 2015; Shukla et al. 2024). Although recently developed transgenic tools allow for more facile manipulation of histone gene copy numbers *in vivo* (Cook et al. 2012; Mckay et al. 2015; Zhang et al. 2019), the tight regulation of histone expression still makes manipulating histone expression levels in *Drosophila* challenging. For example, flies carrying 24 copies of the histone genes have nearly identical levels of histone transcripts and proteins as wildtype flies carrying 200 copies of the histone genes, likely due to a feedback-based compensation mechanism that ensures adequate histone expression levels regardless of histone gene copy number (Mckay et al. 2015). Thus, besides its exciting biology, *ao* emerged as a promising tool for manipulating histone gene expression in *D. melanogaster* (Chari et al. 2019).

Existing *ao* reagents, however, have several caveats. First, only the *ao*^*1*^ strain is still available, whereas the *ao*^***2***^ strain has been lost. Second, the two mutants exhibit different phenotypes: *ao*^***1***^ is viable as a homozygous mutant, but *ao*^***2***^ was reported to be lethal as a homozygote (Tomkiel et al. 1995). Furthermore, although both *ao*^*1*^*/ao*^*1*^ and *ao*^*1*^*/ao*^*2*^ mutants exhibited maternal-effect lethality, more embryos from *ao*^*1*^*/ao*^*1*^ mothers died at earlier stages compared to those from *ao*^*1*^*/ao*^*2*^ mothers (Tomkiel et al. 1995). Finally, *ao*^*1*^*/ao*^*1*^ stocks are unstable and can rapidly acquire genetic suppressors that alleviate the maternal-effect lethality (Krider and Levine 1975; Graziani et al. 1981; Manzi et al. 1986). These observations implied that genetic background effects could dramatically affect the severity of the phenotype associated with loss of *ao*.

To overcome these hurdles and to accurately characterize the *ao* phenotype, we used CRISPR/Cas9-based methods to generate two new *ao* reagents: a precise knockout of *ao* to enable genetic analyses and a V5 epitope-tagged allele of *ao* at the endogenous locus to enable cytological visualization. Using these reagents, we recapitulated several classical genetic and cytological attributes of *ao*, including its maternal-effect lethality, which is suppressed either by a reduction in histone gene copy number or by excess heterochromatin on the Y chromosome. We also found that the Ao protein localizes to the histone gene cluster in ovaries. However, contrary to prior evidence, we discovered that *ao* does not affect histone transcript levels. Unlike in *ao*^*1*^*/ao*^*2*^ flies, histone levels are unaffected in ovaries from both *Δao/Δao* and *ao*^*1*^*/ao*^*1*^ homozygous females. Thus, although *ao* genetically interacts with histones and heterochromatin as proposed in Sandler’s original hypothesis, we conclude that the molecular basis for these interactions remains undiscovered.

## Results

### *Δao*-knockout flies have partial maternal-effect lethality

**Figure 1.**
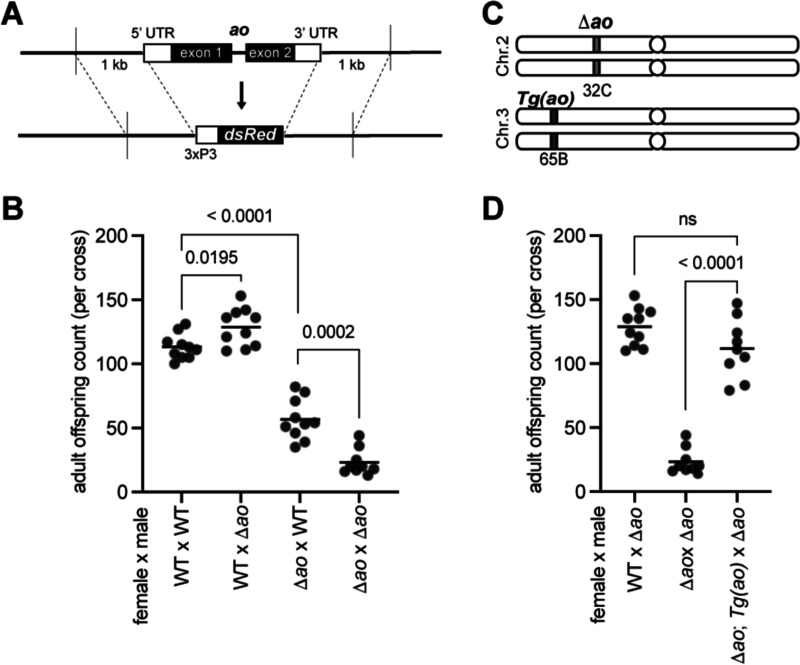
Loss of *ao* causes partial maternal-effect lethality in *D. melanogaster*. **(A)** We replaced the *ao* coding sequence and UTRs with *dsRed* (fluorescent marker) under the control of *3xP3*, an eye-specific promoter, using CRISPR-Cas9 and homology arms (detailed in Figure S1). **(B)** Crosses between Δ*ao* females and wildtype males yield fewer adult progeny than crosses between wildtype females and wildtype males or between wildtype females and Δ*ao* males, confirming that loss of *ao* leads to partial maternal-effect lethality, which is further exacerbated in crosses between Δ*ao* females and Δ*ao* males. The mild increase in offspring number in the cross between wildtype females and Δ*ao* males is not reproducible (Fig. S2). Each point on a graph is the offspring count from a biological-replicate cross performed at 29°C. The *p*-values from two-tailed Mann-Whitney U tests are shown above the compared samples. **(C)** We integrated a transgene *ao* ‘rescue’ construct on the *D. melanogaster* 3^rd^ chromosome using the PhiC31 integrase system (details in Fig. S7). The ‘rescue’ ao transgene is expressed at ~20% of the levels of the endogenous ao gene. **(D)** Despite being expressed at only ~20% of the levels of the endogenous *ao* locus (Fig. S8), the ‘rescue’ *ao* transgene can restore the number of adult offspring produced from Δ*ao* females to nearly wildtype levels at 29°C. The *p*-values are from two-tailed Mann-Whitney U tests.

To obtain an *ao* mutant without genetic background effects, we used CRISPR/Cas9 to create a *Δao* strain using guide RNAs designed to target the start of the 5’ UTR and the end of the 3’ UTR of *ao* (Fig 1A). To facilitate the phenotypic screening of *Δao* flies, we inserted a repair template with the fluorescent marker *dsRed* under the control of the eye-specific promoter *3xP3* using homology arms of approximately 1000 base pairs. We verified the *ao* knockout and *dsRed* replacement using PCR and Sanger sequencing (Fig S1). We also generated a nearly isogenic strain to the *yw; Δao/CyO-gfp* strain except for a wildtype 2^nd^ chromosome in place of *Δao*, which we used as the ‘wildtype’ control for all future experiments.

We first assessed whether our newly generated *Δao* strain recapitulated the maternal-effect lethality phenotype of *ao* mutants (Sandler 1970; Sandler 1977; Tomkiel et al. 1995). Since *ao*-associated maternal-effect lethality was more pronounced at higher temperatures (Sandler 1970), we measured the total number of adult offspring produced by *Δao* flies at 29°C. Consistent with previous findings, we found that *Δao* females exhibit partial maternal-effect lethality when crossed to wildtype males (Fig 1B). We found that maternal-effect lethality is exacerbated in crosses between *Δao* females and *Δao* males (Fig 1B), confirming previous findings that a paternal copy of the wildtype *ao* allele can partially rescue zygotic survival (Pimpinelli et al. 1985; Tomkiel et al. 1995). Although our data initially suggested a slight fertility increase of *Δao* males relative to wildtype males in crosses with wildtype females (Fig 1B), subsequent experiments revealed no significant differences in these crosses (Fig S2).

We confirmed our findings by measuring the survival of pupae or adults from a given number of larvae produced from *ao*^*1*^*/ao*^*1*^ mutant mothers. This assay examines viability at later developmental stages rather than at all stages (Fig 1B). Nevertheless, these findings (Fig S3) were nearly identical to our previous findings of offspring viability from *Δao/Δao* mothers. Thus, the maternal-effect lethality resulting from *ao* loss continues to manifest at both early and later stages of development and can be further exacerbated by the loss of a paternal (and zygotic) *ao*.

We further assessed zygotic effects or survival of *Δao* flies by looking for deviations from the expected Mendelian ratio in offspring genotypes. Without a zygotic effect, the theoretical Mendelian ratio for offspring from two heterozygous parents should be 33% *Δao* homozygotes and 66% heterozygotes (homozygosity for balancer chromosomes leads to lethality). In contrast to this expectation, the observed offspring genotype ratio from parents heterozygous for *Δao* was 28% homozygous and 72% heterozygous, indicating a statistically significant but mild zygotic effect (one-sample proportion test, *p* = 0.0043; Fig S4).

Our findings of *ao*-associated maternal-effect lethality are most pronounced at 29°C but also manifest at 25°C and 18°C, albeit to slightly lower extents (Fig S5), consistent with previous findings (Sandler 1970). Finally, as previously reported, we found that heterozygous females with one copy of the *Δao* allele produce the same number of adult offspring as wildtype females (Fig S6) (Sandler 1970). Thus, the *Δao* strains we created confirm previous findings from *ao*^*1*^ *and ao*^*2*^ strains: loss of *ao* results in no significant consequences to paternal fertility but does cause maternal-effect lethality, which can be partially rescued by a wildtype paternal allele of *ao* in the zygote.

We generated a ‘rescue’ *ao* transgene to rule out the possibility that the maternal-effect lethality in Δ*ao* strains might have resulted from an off-target mutation introduced during the CRISPR/Cas9 cleavage or repair (Fig 1C). We used the PhiC31 integrase system (Groth et al. 2004) to insert the *ao* transgene onto the 3^rd^ chromosome, flanked by approximately 700 base pairs upstream and 300 base pairs downstream of the *ao* protein-coding sequence. Since the *ao* promoter is poorly defined, we included the untranslated regions of neighboring genes but not their coding sequences. By crossing the *Δao* and ‘rescue’ *ao* strains, we obtained flies with homozygous *Δao* mutations (on the 2^nd^ chromosome) with two copies of the ‘rescue’ *ao* transgene (on the 3^rd^ chromosome) (Fig S7). The ‘rescue’ *ao* transgene is transcribed at only 20% of the level of the endogenous *ao* gene in ovaries (Fig S8). Despite its lower expression, the ‘rescue’ *ao* transgene is sufficient to suppress the maternal-effect lethality of *Δao* entirely (Fig 1D), thereby demonstrating a causal association of the maternal-effect lethality we observed with loss of *ao*.

### Ao localizes to the histone gene cluster but does not affect histone transcript levels

**Figure 2.**
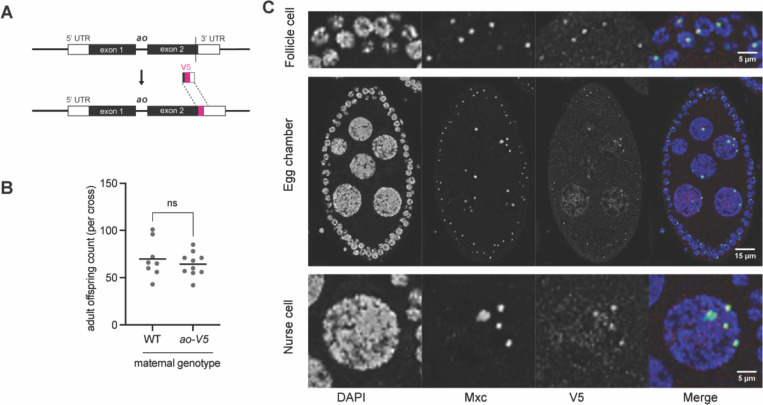
Ao protein localizes to the histone gene locus in *D. melanogaster* ovaries. **(A)** Using CRISPR-Cas9, we introduced an in-frame V5 epitope tag at the 3’ end of the *ao* gene at its endogenous locus (details in Fig. S9). **(B)** Crosses between *ao-V5* females and *yw* males yield the same number of adult offspring as those between *yw* females and males at 29°C. The *p*-values are from two-tailed Mann-Whitney U tests. **(C)** Using immunofluorescence studies in ovaries from *ao-V5* ovaries, we investigated the localization of DNA (visualized using DAPI), Ao-V5, and the MxC protein, which localizes to histone locus bodies. The top row shows a group of somatic follicle cells in which Ao-V5 co-localizes with MxC. (Fig. S10 shows additional representative images of Ao-V5 and MxC co-localization). The merged image is shown in color, with DAPI, Mxc, and V5 in blue, teal, and magenta, respectively. The middle row shows a single egg chamber containing polyploid nurse cells surrounded by somatic follicle cells. The third row shows a single nurse cell nucleus, with clear co-localization of Mxc and Ao-V5 puncta. (Figs. S10-S13 shows additional representative images of Ao-V5 and MxC co-localization, whereas Figs. S14-15 show controls).

Using a polyclonal antibody raised against the Ao protein, a previous study reported localization of Ao to the multigene array of histone genes on the 2^nd^ chromosome in polytene chromosomes from salivary glands and mitotic chromosomes from larval neuroblasts (Berloco et al. 2001). However, this antibody is no longer available. To visualize the Ao protein, we generated an *ao-V5* strain, where the *ao* gene was epitope-tagged at its C-terminus with a V5 epitope at the endogenous locus. We used CRISPR/Cas9 to insert the V5 tag at the 3’ end of the coding region at the endogenous *ao* locus (Fig 2A, S9). We confirmed that the *ao-V5* female flies have wildtype fertility at 29°C, indicating that the V5 tag does not interfere with Ao protein function (Fig 2B). Given *ao*’s maternal-effect lethal phenotype, we assayed the localization of the Ao-V5 protein in ovaries using an antibody raised against the V5 epitope. We found that Ao-V5 colocalizes in the nucleus of ovaries with Multi sex combs (Mxc), which localizes specifically to the histone gene cluster and is a core structural component of the histone locus body (Fig 2C, S10) (White et al. 2011). The Ao-V5 and Mxc co-localization occurs in somatic follicle cells and germline nurse cells. Although Mxc and Ao-V5 puncta colocalize in most cells, we find some Ao-V5 puncta without Mxc, suggesting either additional genomic localization for Ao or differences in the composition of the histone locus body at different cell cycle stages (Fig 2C, S10).

A remarkable finding from a previous study was the significant overexpression of core histone genes in *ao* mutants (Berloco et al. 2001). Based on Northern blotting analyses, the study reported that unfertilized eggs from *ao*^*1*^*/ao*^*2*^ females had a 1.6-fold (for histone H4) to 11-fold (for histone H2A) increase in core histone transcript levels relative to unfertilized eggs from wildtype Oregon-R females (Berloco et al. 2001).

**Figure 3.**
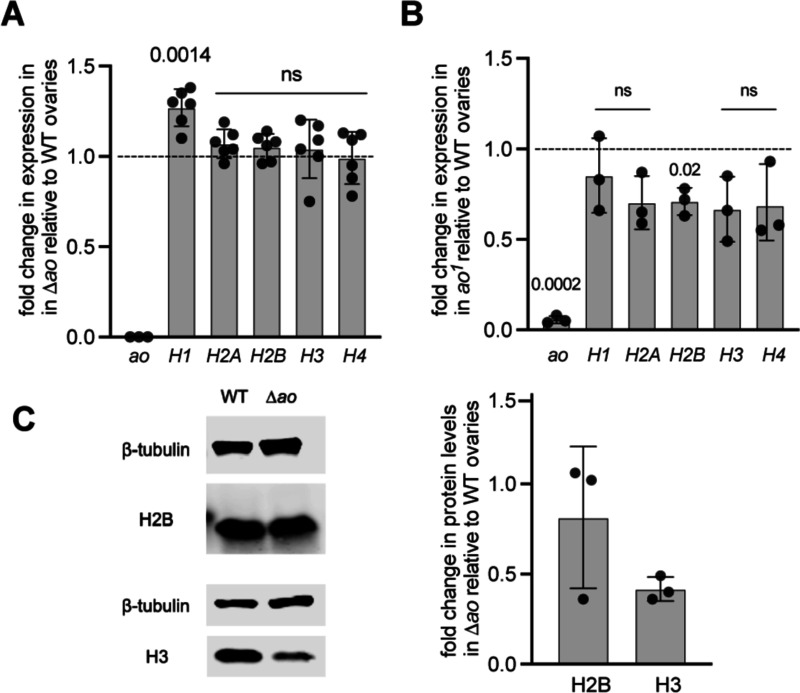
Histone expression levels in *ao* mutant ovaries. **(A)** RT-qPCR on Δ*ao* ovaries presents the levels of the linker (H1) and core (H2A, H2B, H3, H4) histone transcripts relative to isogenic wildtype (isogenic *yw*) females (dashed line). Each data point is a biological replicate of 3–5 ovaries from virgin females. For each replicate, the median of the technical triplicate is shown. Gene expression has been normalized to ribosomal protein *rp49*. The *p*-values are calculated using a one-sample t-test (Methods). **(B)** RT-qPCR on *ao*^*1*^ ovaries presents the levels of the linker (H1) and core (H2A, H2B, H3, H4) histone transcripts normalized to wildtype females (dashed line); we note that we do not have isogenic wildtype females for this comparison. Each data point is a biological replicate of 3–5 virgin ovaries. For each replicate, the median of the technical triplicate is shown. Gene expression has been normalized to *rp49*. The *p*-values are calculated using a one-sample t-test (Methods). **(C)** Western blots on Δ*ao* ovaries reveal no difference in H2B levels between Δ*ao* and wildtype (isogenic *yw*) ovaries, but 50% lower levels of H3 protein in Δ*ao* ovaries. We use beta tubulin as a loading control for visualization and quantification.

To quantify histone transcript levels in ovaries and unfertilized eggs from *Δao* virgin females, we performed quantitative reverse transcriptase PCR (RT-qPCR) for each of the four core histones and the linker histone, using a similar strategy as previously reported (Bulchand et al. 2010; Rieder et al. 2017) but with slightly different primers (see Methods). Surprisingly, we found no significant differences in core histone levels between *Δao* and isogenic-wildtype samples in both ovaries and unfertilized eggs (Fig 3A, S16). Given the discrepancy between our *Δao/Δao* results and previous findings from *ao*^*1*^*/ao*^*2*^ flies, we further quantified histone transcript levels in *ao*^*1*^*/ao*^*1*^ ovaries by RT-qPCR (the *ao*^*2*^ stock no longer exists and cannot be assayed). Like *Δao* ovaries, we found no evidence for a significant increase in core histone transcript levels in ovaries from *ao*^*1*^*/ao*^*1*^ and wildtype females (Fig 3B). In contrast, we observed a mild but statistically significant decrease in core histone H2B transcripts. Similarly, when assessing core histone transcript levels in unfertilized eggs from *ao*^*1*^*/ao*^*1*^ females, we again observed either no difference or a slight decrease (for histone H2B) in core histone transcript levels (Fig S16). Thus, contrary to the previously published study, we conclude that loss of *ao* does not increase histone gene expression at the steady-state RNA level.

Although this was not explored in the original study (Berloco et al. 2001), we considered the possibility that *ao* might affect histone levels post-transcriptionally. Previous analyses had found that histone H2B levels are increased approximately twofold in embryos resulting from *ao*^*1*^*/ao*^*1*^ females, whereas H3 levels were unchanged (Chari et al. 2019). To investigate this possibility, we quantified the amounts of H2B and H3 proteins in *Δao* and isogenic-wildtype ovaries using western blotting analyses. We found no evidence for increased histone protein levels in *Δao* ovaries (Fig 3C). Instead, we found a slight decrease in H3 protein levels (Fig 3C). Thus, our RT-qPCR and western blotting analyses conclude that loss of *ao* does not significantly increase histone transcript or protein levels in ovaries, challenging previous conclusions that Ao acts as a repressor of core histone expression (Berloco et al. 2001).

### Genetic interactions between *ao*, histone genes, and Y-chromosomal heterochromatin

**Figure 4.**
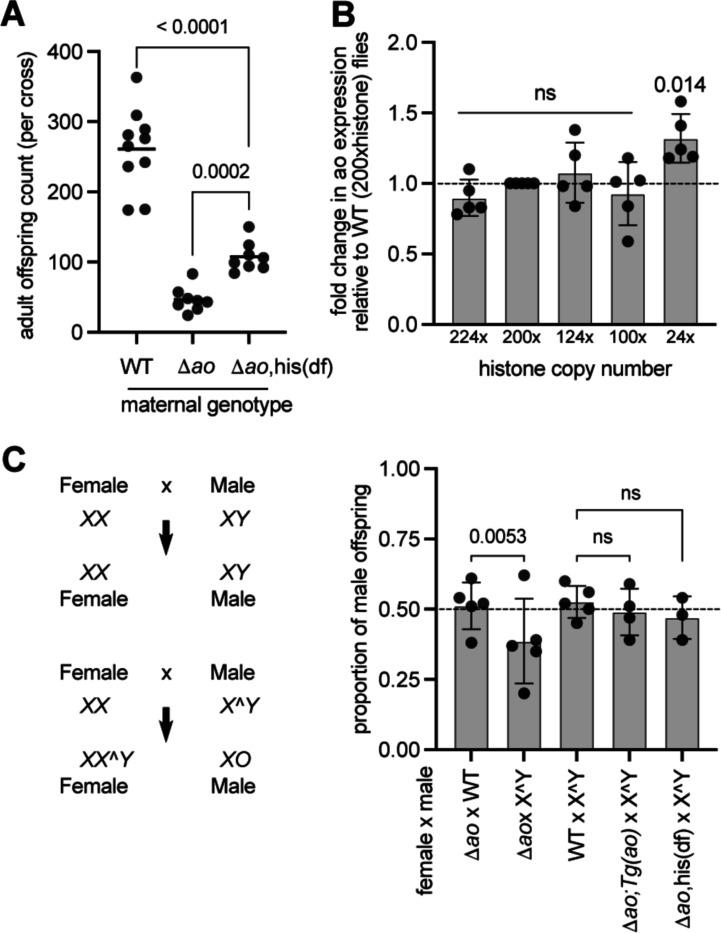
Histone deficiency and Y-chromosome heterochromatin ameliorates *ao’*s maternal-effect lethality. **(A)** We compared the total number of offspring produced by wildtype (isogenic *yw*) females, Δ*ao* females, and Δ*ao* females harboring a heterozygous histone deficiency (his(df), which has a deletion from cytological locus 39D3 to 39E2, corresponding to the histone gene array, Fig. S17) in crosses to Δ*ao* males at 29°C. Each point on the graph is the offspring count from a biological-replicate cross. The *p*-values are from two-tailed Mann-Whitney U tests. **(B)** Using RT-qPCR, we measured *ao* transcript levels in flies carrying different numbers of histone genes (Fig. S18). Each data point is a biological replicate of 4 virgin ovaries. The mean of the biological replicate is shown for each replicate. Gene expression has been normalized to *rp49*. The *p*-values are calculated using a one-sample t-test (Methods). **(C)** Adult offspring are produced in an equal sex ratio (50% male) in crosses between Δ*ao* females and wildtype males or crosses between wildtype females and attached X^Y males (BDSC strain 9460) at 25°C. In contrast, in crosses between Δ*ao* females and males carrying an attached X^Y sex chromosome, adult progeny counts are skewed to produce more XX^Y females relative to XO males at 25°C. However, this sex-ratio skew is rescued either by the presence of two alleles of the *ao* ‘rescue transgene’ on the 3^rd^ chromosome (Fig. 1C) or by the histone deficiency on the 2^nd^ chromosome (Fig. 4A). The *p*-values are from a 2×2 contingency table using the two-tailed Fisher’s exact test.

The previous finding that *ao* might encode a histone gene repressor motivated the hypothesis that *ao*-associated maternal-effect lethality results from excess histones in eggs (Berloco et al. 2001). This hypothesis led to the prediction that *ao*-associated maternal-effect lethality might be rescued by a histone deficiency in *ao*-mutant mothers. Indeed, a hemizygous deletion of the histone locus in *ao*^*1*^/*ao*^*1*^ mutant females was shown to ameliorate their maternal-effect lethality (Berloco et al. 2001). Although we found no evidence of *ao* acting as a histone gene repressor, we investigated whether a histone deletion could suppress the maternal-effect lethality of *Δao* females. For this, we crossed *Δao* flies into a strain carrying a hemizygous deletion of the histone locus (BDSC 8670) (Cook et al. 2012) to create *Δao/Δao* flies with half the number of histones (Fig 4A, S17). We found that reducing histone copy number partially rescues the maternal-effect lethality of *Δao* females (Fig 4A), just as previously reported for *ao*^*1*^/*ao*^*1*^ females (Berloco et al. 2001).

We also assessed the relationship between histone gene copy number and *ao* expression using flies with a deletion of the histone gene complex (Gunesdogan et al. 2010) as well as a ‘12xhistone’ transgene carrying only twelve copies of the histone genes inserted on the 3^rd^ chromosome (Fig 4B) (Mckay et al. 2015). Although wildtype diploid flies encode 200 copies of histone genes, only twelve copies (*i.e.*, flies encoding only a hemizygous 12xhistone transgene) can remarkably suffice for viability (Gunesdogan et al. 2010; Mckay et al. 2015). If *ao* were functioning as a histone repressor, we hypothesized that flies with such drastically reduced histone copy number would have lower *ao* levels to facilitate higher histone gene expression. We took advantage of endogenous histone locus deletions and 12xhistone transgenes to generate flies encoding 224, 200 (wildtype), 124, 100, or 24 copies of the histone gene cluster (Fig S18). Upon quantifying *ao* transcript levels via RT-qPCR, we found that flies encoding 24 copies of histone genes had a nearly 20% increase in *ao* transcript levels compared to flies encoding 100, 124, 200 (wildtype), or 224 copies of the histone genes (Fig 4B). Our findings are consistent with data from a previous study, which compared the transcriptomes of 200xhistone and 12xhistone flies (Mcpherson et al. 2023). Although it was not the focus of the previous study, this dataset revealed that *ao* transcript levels were slightly higher in animals with fewer copies of the histone genes. Together, these findings run counter to the expectation that *ao* negatively regulates histone gene expression.

Next, we focused on genetic interactions between *ao* and heterochromatin encoded on sex chromosomes. Although *ao* mutant females produce fewer offspring than wildtype females due to their maternal-effect lethality, the offspring are not skewed in their sex ratio. However, *ao* mutant females produce an excess number of XX^Y female offspring relative to X0 male offspring when crossed to males with attached X^Y chromosomes (Sandler et al. 1968; Sandler 1970; Parry and Sandler 1974; Sandler 1977). This distortion of offspring sex ratio is attributed to the ability of specific regions of the X- and Y-heterochromatin to partially relieve *ao*-associated maternal effect lethality (Sandler 1970; Parry and Sandler 1974; Sandler 1977; Yedvobnick et al. 1980; Pimpinelli et al. 1985; Tomkiel et al. 1991).

We confirmed the interaction between the *ao* gene and AO heterochromatin on the Y chromosome using the *Δao* strain. Crosses between *Δao* females and wildtype males yielded nearly equal numbers of female (51%) and male (49%) offspring (Fig 4C). In contrast, crosses between *Δao* females and attached X^Y males (BDSC 9460) yielded a 61:39 female: male offspring ratio, significantly deviating from the 50:50 expectation (2×2 contingency table, two-tailed Fisher’s exact test *p*-value=0.0053). This skew in sex ratio was corrected in crosses between *Δao* females carrying two copies of the 3^rd^ chromosome ‘rescue’ *ao* transgene and attached X^Y males (49% males). Similarly, crosses between *Δao* females carrying a histone deficiency and attached X^Y males also yielded a nearly equal sex ratio among its progeny (47% males) (Fig 4C). Thus, despite our finding that *ao* is not a transcriptional repressor of core histone transcription, we confirmed previous findings that its loss results in maternal-effect lethality, which can be rescued either by depleting histone gene copy number or by Y chromosome-linked heterochromatin.

## Discussion

Pioneering genetic studies identified *ao* as a maternal-effect lethal gene whose maternal-effect lethality depended on heterochromatin content in zygotes (Sandler et al. 1968; Sandler 1970; Parry and Sandler 1974; Sandler 1977). A satisfying explanation for *ao*’s connection to heterochromatin emerged from a molecular study three decades after its initial characterization, which showed that the *ao*-encoded protein localized to the histone gene cluster and that its loss led to histone overexpression in unfertilized eggs from *ao*^*1*^*/ao*^*2*^ mothers (Berloco et al. 2001). The same study also demonstrated that reducing histone copy number ameliorated *ao*-associated maternal-effect lethality. These observations led to the model that histone overproduction in *ao/ao* mothers leads to high histone levels in eggs and zygotes, which results in the maternal-effect lethal phenotype. As a result of these findings, *ao* became a potential tool for manipulating histone gene expression in *Drosophila* (Chari et al. 2019), which cannot be accomplished by simply changing the histone gene copy number (Mckay et al. 2015; Zhang et al. 2019).

However, these observations relied on reagents that have associated genetic background effects. Moreover, two critical reagents – the *ao*^*2*^ allele (Tomkiel et al. 1995) and the anti-Ao antibody (Berloco et al. 2001) – are no longer available. We developed two novel tools to bridge this gap: a precise CRISPR/Cas9-mediated deletion of the *ao* locus (and replacement with *dsRed*) and an *ao* allele tagged with the V5 epitope at the endogenous locus. Using these reagents, we revisited key findings from the original *ao* studies. We confirmed *ao*-associated maternal-effect lethality and its amelioration via a reduction in histone copy number or excess Y-heterochromatin. We also confirmed the localization of the Ao protein to the histone cluster in ovaries. However, we found that *ao* is not a direct repressor of histone expression; for most histone genes, we observed no differences in histone RNA levels among *Δao/Δao*, *ao*^*1*^*/ao*^*1*^, and wildtype ovaries. Our results suggest *ao* should not be used to manipulate histone levels. An alternative tool might be the histone chaperone Nuclear Autoantigenic Sperm Protein (NASP), which is an H3-H4 chaperone in *Drosophila* embryos (Tirgar et al. 2023).

Our findings challenge the previously proposed model that histone overexpression results in *ao*’s maternal-effect lethality (Berloco et al. 2001). Since the *ao* study was published, subsequent studies have demonstrated that dramatically reducing histone gene copy number does not decrease histone expression (Mckay et al. 2015; Mcpherson et al. 2023). These findings also challenge the previous model that decreased histone gene expression must have compensated for histone overexpression in the absence of *ao*. Since *Δao* alleles fully recapitulate the maternal-effect lethality reported in *ao*^*1*^*/ao*^*1*^ and *ao*^*1*^*/ao*^*2*^ females but not the histone overexpression reported in *ao*^*1*^*/ao*^*2*^ trans-heterozygote females, we conclude that histone overexpression is not the mechanism underlying *ao*-associated maternal-effect lethality.

Why is there a significant discrepancy in the histone overexpression phenotype between our *Δao* alleles and the previously characterized *ao*^*1*^*/ao*^*2*^ strain? Precise deletion null alleles (like *Δao*) can often differ in phenotype from presumed null alleles that leave part of the gene intact (like *ao*^*1*^, which has a Doc transposon interrupting the first exon). This difference can result from transcriptional adaptation (El-Brolosy and Stainier 2017), a phenomenon in which alleles that ablate mRNA transcription entirely can get compensated and, therefore, give less severe phenotypes than those that allow transcription of mutant mRNA (El-Brolosy et al. 2019).

However, we suspect the correct explanation might stem from differences in genetic background among *ao* strains. The recent discovery that histone copy number fluctuates up to five-fold among Drosophila strains (Shukla et al. 2024) could partially explain differing effects of *ao* alleles on histone levels in different genetic backgrounds ((Berloco et al. 2001; Chari et al. 2019)(this study)). Like in *Δao*/*Δao* ovaries, we did not observe a histone overexpression phenotype in *ao*^*1*^*/ao*^*1*^ ovaries. Before our present study, *ao*^*1*^*/ao*^*1*^ flies had never been assessed for histone-transcript levels. Indeed, the overexpression of histone mRNAs was only previously reported for the *ao*^*1*^*/ao*^*2*^ trans-heterozygote (Berloco et al. 2001). Unlike *Δao* and *ao*^*1*^ alleles, the *ao*^*2*^ allele could not be made homozygous (Tomkiel et al. 1995), indicating the presence of one or more recessive lethal mutations. Moreover, whereas a wildtype *ao* transgene rescues the maternal-effect lethality of *ao*^*1*^ (Tomkiel et al. 1995) and *Δao* (Fig. 1), a wildtype transgene rescue for histone overexpression or lethality of the *ao*^*1*^/*ao*^*2*^ trans-heterozygote was never reported (Berloco et al. 2001). These observations suggest that the genetic background of the *ao*^*2*^ allele might have conferred an additional phenotypic burden not directly related to *ao* function. For example, *P*-element insertion in the 5’UTR of *ao* in the *ao*^*2*^ strain might have inadvertently affected the expression of the upstream *ATPsynG* gene, which encodes an essential subunit of the mitochondrial ATP synthase subunit (Fukuoh et al. 2014). Unfortunately, since the *ao*^*2*^ strain is no longer available, we cannot test our hypothesis of additional background effects or how they might relate to histone overexpression.

Ao belongs to the DET1 family of E3 ubiquitin ligases (Berloco et al. 2001). Orthologs of Ao include the human *hDET1*, a negative regulator of a proto-oncogene (Wertz et al. 2004; Pick et al. 2007), and the *Arabidopsis DET1*, which functions as a negative regulator of light-mediated growth in seedlings (Chory et al. 1989; Chory and Peto 1990; Pepper et al. 1994). *Arabidopsis* DET1 binds to nonacetylated, C-terminal H2B tails in the nucleosome (Benvenuto et al. 2002) and regulates H2B mono-ubiquitination in a light-dependent context (Nassrallah et al. 2018). Given the conservation of the plant and mammalian DET1 proteins as subunits of the COP1 Cul4A-RING E3 ubiquitin ligase complex (Wertz et al. 2004; Yanagawa et al. 2004; Bernhardt et al. 2006; Pick et al. 2007), it is highly likely that *Drosophila* Ao also functions in post-translational, rather than transcriptional, regulation. However, our western blotting analyses suggest that histones are not the post-translational target of *ao*.

Our findings suggest that a different molecular mechanism underlies *ao* function and its genetic interactions with the histone gene cluster and heterochromatin. We speculate that *ao* function might be affected by some unknown component of the histone locus body, which regulates histone biogenesis (White et al. 2011; Duronio and Marzluff 2017). In this model, loss of *ao* could be compensated by simultaneous depletion of this unknown component via reduction or loss of the histone locus. Alternatively, the histone gene cluster might be a sink for another factor essential for embryonic viability in the absence of *ao*. When histone gene dosage is reduced, this factor is freed to relieve maternal-effect lethality caused by loss of *ao*. Under this model, *D. melanogaster* strains that naturally encode high histone gene copy number (Shukla et al. 2024) might be prone to more severe maternal-effect lethality upon loss of *ao*, whereas strains that naturally encode fewer genes or those carrying a histone deficiency might be better able to withstand the loss of *ao*. Although our findings challenge the current molecular model of *ao*, these possibilities highlight that understanding the basis of *ao*-associated maternal-effect lethality and its connection to histone copy number and heterochromatin remains an open and exciting question.

## Materials and Methods

### Generation of the *Δao* line

We used CRISPR/Cas9 to create an *ao* knockout line. To facilitate the screening of transgenic flies, we replaced the *ao* allele with *dsRed* under the eye-specific promoter *3xP3*. We chose guide RNAs with the best efficiency score and no predicted off-targets (https://www.flyrnai.org/crispr/). We cloned guide RNAs (AGCCGGGTTCTTCTTCCGAT and AGTAATGTCTTTATTTACAA) targeting the 5’ and 3’ ends of the *ao* gene into pCFD4 U6:1_U6:3tandemgRNAs (Port et al. 2014; Addgene plasmid #49411). The repair template sequence, including homology arms spanning approximately 1kb upstream and downstream of the *ao* coding sequence, was cloned into pDsRed-attP (Gratz et al. 2014) (Addgene plasmid #51019). To prevent guide RNAs from targeting PAM sites, we mutated the PAM sites using the Q5 Site-Directed Mutagenesis Kit (New England Biolabs).

BestGene Inc. (Chino Hills, CA) prepared and co-injected the plasmids into BDSC 51323 embryos expressing *vas-Cas9* on the X chromosome. Following injection, BestGene Inc. crossed the injected flies to a *yw* strain to isolate transformants, crossed out the *Cas9* gene, and balanced the 2^nd^ chromosome over *CyO*. We verified the absence of *ao* and the presence of *dsRed* with PCR and Sanger sequencing (see Table S1 for primer sequences). We extracted genomic DNA with the DNeasy Blood & Tissue Kit (Qiagen) according to the manufacturer’s protocol for insect tissue. We performed PCR using the Platinum PCR SuperMix High Fidelity (Invitrogen). The penetrance of the *CyO* phenotype decreased with temperature, which made it difficult to distinguish between homozygote-null and heterozygous flies. We, therefore, rebalanced the *Δao* allele over *CyO-gfp* marked with *mini-white*, which enabled us to screen for homozygous flies based on eye pigment color. To allow for *mini-white* visualization, the *CyO-gfp* strain carries *yw* on the X chromosome. We crossed the *yw* strain from BestGene with our *yw; CyO-gfp* strain to obtain a near-isogenic strain to our *Δao* strain and used this strain as the wildtype control for all experiments.

To obtain an *Δao* strain with a hemizygous histone deletion, we crossed our *Δao* flies into the BDSC 8670 strain (Cook et al. 2012), which has a heterozygous deletion on the 2^nd^ chromosome corresponding to the histone gene array (chromosomal locus 39D3 to 39E2). Both the *ao* allele and histone genes are located on the left arm of the 2^nd^ chromosome, so after obtaining a female fly heterozygous for both *Δao* and histone deficiency, we relied on recombination to obtain a fly with *Δao* and histone deficiency on the same 2^nd^ chromosome. We used this fly to make the *Δao, his(df)* stock. The histone deletion is not marked, so we used PCR using the Phusion High-Fidelity DNA polymerase (New England Biolabs) to determine its presence in the founder fly (see Table S1 for primer sequences).

### Generation of the *ao*-transgene rescue line

We used the PhiC31 integrase system to create the “rescue” line with wildtype *ao*. We cloned the *ao* coding sequence with its endogenous promoter into the pattB plasmid containing an *attB* site and *mini-white* marker (Bischof et al. 2013)(DGRC stock 1420). BestGene Inc. prepared and injected the plasmid into BDSC 9750 embryos, which have the VK33 *attP* landing site on the 3^rd^ chromosome (Venken et al. 2006). BestGene Inc. confirmed successful integration with PCR to verify the presence of the recombined *attL* site and absence of the original *attP* site (see Table S1 for primer sequences), then crossed out the gene encoding the PhiC31 integrase and balanced the 3^rd^ chromosome over *TM6B*.

### Generation of the V5-tagged *ao* line

We used CRISPR/Cas9 to tag *ao* at its C-terminus with a V5 epitope. We chose a guide RNA closest to the stop codon of *ao* with no off-targets (http://targetfinder.flycrispr.neuro.brown.edu/). We cloned the guide RNA (GTATAACCACAGCACAATAG) into pCFD5 (Port and Bullock 2016)(Addgene plasmid #73914). We designed a single-stranded oligo donor (ssODN) repair template containing the V5 tag (42 bp) and approximately 55 bp up- and downstream of the insertion site. The ssODN had a mutated PAM site to prevent re-targeting.

We sent the midi-prepped pCFD5 plasmid containing the guide RNA and lyophilized ssODN to GenetiVision Inc. (Houston, TX) for injection into embryos expressing *nanos*-*Cas9*. We screened for transformants using a PCR strategy, with primers that annealed upstream and downstream of the insertion site (see Table S1 for primer sequences). We tested for insertion of the V5 tag by the presence of a 42 bp shift in band size. Finally, we confirmed the successful insertion of the intact V5 tag by Sanger sequencing.

### Fly husbandry, fertility, and viability assays

Flies were maintained on the benchtop at room temperature on corn syrup/soy media made in-house at Fred Hutch Cancer Center (Seattle, WA) or purchased from Archon Scientific (Durham, NC). To conduct fertility assays, we used 1-to-5-day-old males and virgin females raised at room temperature. Unless otherwise noted, we paired four virgin females with two males in a vial with corn syrup/soy media and allowed them to mate for three days (for X^Y assays) or one week (for all other assays). To prevent larval overcrowding in the vials, we flipped the parents to new vials after three days and discarded the parents from the new vials four days later. As noted for each experiment, the crosses were set up and maintained at 18°C, 25°C, or 29°C. We counted the adult offspring (F1) to exhaustion, *i.e.*, until no more progeny were produced.

We excluded crosses with no larvae in one or both vials from statistical analyses. Because non-genetic factors, including variation in food and ambient humidity, influence fly fertility, we only compared data among crosses set up on the same day. We used GraphPad Prism version 10.1.1 for macOS (GraphPad Software) to plot the data and conduct statistical analyses. We performed two-tailed Mann-Whitney U tests to compare the offspring count between the two datasets and reported exact *p*-values. To compare the observed offspring genotype to the theoretical Mendelian offspring genotype in Fig S2, we used a one-sample proportion test (http://www2.psych.purdue.edu/~gfrancis/calculators/proportion_test_one_sample.shtml). To compare the results of X^Y crosses in Fig 4C, we analyzed a 2×2 contingency table using a two-tailed Fisher’s exact test (https://www.graphpad.com/quickcalcs/contingency1/).

We performed late-stage developmental viability assays as previously described (Spring et al. 2019). Briefly, we transferred 40–50 second-instar larvae into vials containing standard molasses fly food and waited for them to complete development. We counted the number of pupal cases in each vial and the number of eclosed adults. We calculated the percentage viability at pupal and adult stages by dividing these values by the initial number of larvae and multiplying by 100. Each vial is one biological replicate, and we had 5–8 replicates for each genotype.

### Immunofluorescence

We dissected ovaries in PBS, then fixed with 1:1 paraPBT: heptane (paraPBT = 4% paraformaldehyde in PBS + 0.1% Triton X-100) in an Eppendorf tube for 10 minutes at room temperature. Following three 5-minute washes in PBST (PBS + 0.1% Triton X-100), we blocked the ovaries in PBST with 3% BSA for 30 minutes at room temperature. We incubated the ovaries in primary antibodies overnight at 4°C. We used a guinea pig anti-Mxc (gift from Robert Duronio) at a 1:5000 dilution and the V5 Tag monoclonal antibody (Thermo R960–25) at a 1:250 dilution. After three 5-minute washes in PBST, the samples were incubated with secondary antibodies in PBST for 2 hours at room temperature. We used the goat anti-mouse IgG Alexa Fluor 568 (Thermo A-11031) and goat anti-guinea pig IgG Alexa Fluor 488 (Thermo A-11073), both at 1:2000 dilution. Hoechst stain (Invitrogen) was added to the samples in the last 30 minutes of the incubation with the secondary antibody. After three 5-minute washes with PBST, the ovaries were mounted onto slides with 20µL of SlowFade Gold Antifade Mountant with DAPI (Invitrogen), and then coverslips were added and sealed with nail polish.

### RNA extraction and RT-qPCR

We dissected Δ*ao* or *ao*^*1*^ ovaries from 4-day-old virgin females in PBS. To collect unfertilized eggs, we let 3-to-7-day-old virgin *ao*^*1*^*/ao*^*1*^ females lay on grape plates for 7 hours. We transferred four pairs of ovaries or ten unfertilized eggs to an Eppendorf tube and homogenized the tissue in 20µL of TRIzol (Invitrogen) with a disposable pestle and electric homogenizer. The samples were stored at −80°C in 100µL of TRIzol until ready to be processed. We incubated the thawed samples in 1mL of TRIzol for 5 minutes, then centrifuged at 13,000rpm for 10 minutes at 4°C to separate the supernatant. We extracted the supernatant with chloroform and extracted the soluble phase with isopropanol. After a wash in 70% ethanol, we resuspended the RNA pellet in RNAse-free water. The samples were treated with DNAse I (Zymo Research) and then purified with the RNA Clean & Concentrator-5 kit (Zymo Research). We quantified the purified samples with the Qubit RNA Broad Range Assay Kit (Invitrogen), then synthesized cDNA using the SuperScript III First-Strand Synthesis System with random hexamers (Invitrogen).

To perform RT-qPCR on Δ*ao* or *ao*^*1*^ ovaries, we used the PowerUp SYBR Green Master Mix for qPCR (Applied Biosystems) with approximately 10ng of cDNA per reaction. We used the QuantStudio 3 Real-Time PCR System (Applied Biosystems) to run the RT-qPCR experiment (see Table S1 for primer sequences). We ran each sample in technical triplicates for each primer pair and used the median value for analysis. We normalized gene expression to the reference gene *rp49* and calculated fold change using the 2^–ΔΔ^Ct^^ method (Livak and Schmittgen 2001). We used the one-sample t-test to compare the *ao* mutant fold change (normalized to *rp49* expression) to the wildtype control.

To measure histone transcript levels in unfertilized eggs from *ao*^*1*^*/ao*^*1*^ homozygous mothers, we used a slightly different set of primers (see Table S1) and AzuraQuant Green Fast qPCR Mix LoRox (Azura genomics). We report a mean of 2 technical duplicates and 4–5 biological replicates. We used the same setup to measure *ao* transcript levels in *D. melanogaster* strains with different histone copy numbers (Fig 4B, primers in Table S1).

### Western blotting

We dissected ovaries from four-day-old virgin females in PBS on ice. We transferred five pairs of ovaries to an Eppendorf tube, added 20µL of 2x Laemmli Buffer (Bio-Rad Laboratories) with 200 mM DTT and 300 mM NaCl, and flash-froze the samples in liquid nitrogen. The samples were stored at −80°C until they could be processed. To extract protein, we thawed the samples on ice and added protease inhibitors (EDTA-free cOmplete ULTRA Tablets, Roche), then hand-pestled the samples on ice using disposable pestles. After a brief spin at 4°C to collect the samples, we boiled the samples at 100°C for 10 minutes, then centrifuged the samples at maximum speed for 2 minutes to obtain clean lysate.

We loaded 10µL of protein sample per well on the Any kD Mini-PROTEAN TGX Precast Protein Gel (Bio-Rad Laboratories). We ran the gel for 90 minutes at 100V in Tris/Glycine/SDS buffer, then transferred the gel to a Trans-Blot Turbo Mini 0.2µm Nitrocellulose membrane (Bio-Rad Laboratories) using the Trans-Blot Turbo Transfer System (Bio-Rad Laboratories). The membrane was washed three times with PBS, blocked in Intercept (PBS) Blocking Buffer (LI-COR) for 1 hour at room temperature, then probed with primary antibodies in phosphate-buffered saline with 0.1% Tween-20 (PBST) at 4°C overnight. We used the following primary antibodies: rabbit anti-beta tubulin (abcam 6046) at 1:1000 dilution, mouse antiH2B (abcam 52484) at 1:3000 dilution, and rabbit anti-H3 (abcam 1971) at 1:4000 dilution. Following three 10-minute washes in PBST, the membrane was incubated for 1 hour at room temperature with IRDye 680RD Donkey anti-Mouse IgG (LI-COR) and/or IRDye 800CW Donkey anti-Rabbit IgG 800 (LI-COR) secondary antibodies in PBST at 1:20,000 dilution. After three washes with PBST and a final wash in PBS, we scanned the membrane at 700nm and 800nm on the Odyssey CLx Imager (LI-COR). We quantified the blots using Image Studio v6.0 (LI-COR). We used the Manual analysis option with median background correction (border 3 for all segments). For each pair of samples, we used the “Add Rectangle” function to make a box around the larger band, then added the same-sized box to the other band using the “Add Selection” function. We normalized H2B or H3 expression to beta-tubulin expression as a loading control, then normalized the Δ*ao* sample to the corresponding wildtype (*yw*) sample to obtain relative quantification.

## Supplementary Material

Supplement 1

Supplement 2

Supplement 3

Supplement 4

Supplement 5

Supplement 6

Supplement 7

Supplement 8

Supplement 9

Supplement 10
