# Supplementary material for: The Drosophila maternal-effect gene abnormal oocyte (ao) does not repress histone gene expression
Source: bioRxiv. 2024 Sep 18:2024.09.17.613536. Preprint. [Version 1] doi: 10.1101/2024.09.17.613536 (PMC11429765; doi:10.1101/2024.09.17.613536)
Supplement: Supplement 1 [file media-1.docx]

**Supplementary materials**

**Supplementary Table S1. List of Primer sequences.**

**Supplementary Table S2. Details of fertility assays reported in the paper.**

**Supplementary Table S3. Details of RT-qPCR analyses reported in Fig.3A.**

**Supplementary Table S4. Details of RT-qPCR analyses reported in Fig.3B.**

**Supplementary Table S5. Quantification of western blot analyses reported in Fig. 3C.**

**Supplementary Table S6. Details of RT-qPCR analyses reported in Fig.4A.**

**Supplementary Table S7. Details of RT-qPCR analyses reported in Fig. S8.**

**Supplementary Table S8. Details of RT-qPCR analyses reported in Fig. S16.**

**Supplementary Figure S1.** **CRISPR/Cas9-mediated *ao* knockout.** We replaced the *ao* coding sequence and UTRs with *dsRed* (fluorescent marker) under the control of *3xP3*, an eye-specific promoter*.* The repair template sequence included homology arms spanning approximately 1kb upstream and downstream of the *ao* coding sequence. We confirmed the *ao* (1851 bp) knockout and *3xP3-dsRed* (903 bp) replacement with PCR using two sets of primers. The first primer set 1 (F1, R1) is external to the repair template. The expected size for the original wildtype *ao* gene is 4139 bp, whereas it is 3191 bp for the *dsRed* replacement (heterozygous flies have both bands). The expected wildtype band for primer set 2 (F2, R2), within the *ao* coding sequence, is 883 bp. This band is absent in the homozygous knockout flies.

**Supplementary Figure S2. No evidence for paternal-effect lethality or enhancement in ∆*ao* flies.** We set up crosses between wildtype (Oregon R) females and wildtype (*yw*) or *Δao* males at 25°C, using three females and one male per replicate vial. The *p-*values are from two-tailed Mann-Whitney U tests.

**Supplementary Figure S3. Late-development stage viability assays for progeny from *ao^1^* females*.* (A)** Pupal viability was measured as a percentage of surviving pupae from larvae resulting from crosses between *yw* females and males, or wildtype females and *ao^1^* males, or *ao^1^* females and wildtype males, or between *ao^1^* females and *ao^1^* males. **(B)** Similar to (A), we measured total adult survival as a percentage of larvae in each of the four crosses. These results can be compared to the total viability results (cumulative from all developmental stages) from *Δao* females (Fig. 1B).

**Supplementary Figure S4. Zygotic effects in ∆*ao* flies.** We crossed *Δao/CyO-GFP* heterozygous females and males to each other at 25°C*.* Since resulting *Cyo-GFP/CyO-GFP* homozygous zygotes do not survive, we expect a ratio of 33% *Δao/Δao* homozygotes among surviving adult progeny. In contrast to this expectation, we find a slightly lower recovery of *Δao/Δao* among surviving adult progeny, indicating a mild but statistically significant zygotic effect. The *p*-value is from the one-sample proportion test.

**Supplementary Figure S5. Temperature-dependence of *ao*-associated maternal-effect lethality.** We set up crosses between ∆*ao* females and wildtype (Oregon R) males or between wildtype females and males at 18°C, 25°C, and 29°C, with five females and two males per replicate vial. Although maternal-effect lethality is apparent at each temperature condition, it is most pronounced at 29°C.

**Supplementary Figure S6. Fertility of heterozygous ∆*ao* females.** To assess whether loss of *ao* causes a dosage-dependent maternal-effect lethality, we crossed either wildtype (isogenic *yw*), *∆ao/+* heterozygous, or *∆ao/∆ao* females to *∆ao/∆ao* males at 29°C. The total number of adult offspring produced was indistinguishable between homozygous wildtype and *∆ao/+* heterozygous female parents. The *p-*values are from two-tailed Mann-Whitney U tests.

**Supplementary Figure S7. Constructing an *ao* ‘rescue’ transgene.** The *ao* coding sequence and ~700bp upstream and ~300bp downstream sequence were inserted on the 3^rd^ chromosome using the PhiC31 integrase system. The absence of the endogenous *ao* allele (and replacement with *dsRed*) on the 2^nd^ chromosome (Fig. S1), the presence of the *attL* site on the 3^rd^ chromosome, and the presence of the *ao* ‘rescue’ transgene on the 3^rd^ chromosome were confirmed with PCR. The expected wildtype *ao* and knockout replacement *dsRed* bands on the 2^nd^ chromosome are 4139 bp and 3191 bp, respectively (Primer Set 1, Fig. S1). Flies carrying an *attL* site have an expected band size of 700bp, which is present in ∆*ao* flies carrying the *ao* transgene on the 3^rd^ chromosome but missing in both wildtype and ∆*ao*. Wildtype flies, and ∆*ao* flies carrying the *ao* transgene on the 3^rd^ chromosome also have a wildtype band (883 bp) for *ao,* the primers for which lie within the *ao* coding sequence (Primer set 2, Fig. S1); this band is missing in ∆*ao* flies not carrying the *ao* transgene.

**Supplementary Figure S8. Expression levels of the *ao* ‘rescue’ transgene.** We performed RT-qPCR on ovaries from virgin ∆*ao,* ∆*ao;tg* homozygotes (with two copies of the *ao* ‘rescue’ transgene)*,* and wildtype (isogenic *yw*) females. These experiments reveal that the *ao* rescue transgene is only expressed to 20% of the levels of the endogenous *ao* locus. Each data point is a biological replicate of 4 virgin ovaries. For each replicate, the median of the technical triplicate is shown. Gene expression has been normalized to *rp49* (data in Table S7).

**Supplementary Figure S9. Schematic of the CRISPR/Cas9-mediated insertion of the V5 tag** **on the *ao* 5’ end.** We used a CRISPR/Cas9-mediated incision at the *ao* 3’ end and a single-stranded oligo donor repair template containing the V5 tag (42 bp) and approximately 55 bp upstream and downstream of the insertion site to introduce the V5 tag in frame with the 3’ end of the *ao* coding sequence. This resulted in a 14 amino acid residue V5 tag at the carboxy-terminal end of the encoded Ao protein. The V5-tag insertion was confirmed with PCR. The expected bands are 170 bp for wildtype (no V5 tag) and 212 bp with the V5 tag.

**Supplementary Figure S10-S13. Ao-V5 colocalization with Mxc in ovaries.** Either follicle cell or nurse cell nuclei are stained with DAPI (blue in merged image), and antibodies to Mxc (Multi sex combs protein, which localizes to the histone locus body, teal in merged image) and V5 (staining Ao-V5, magenta in merged image).

**Supplementary Figure S14.** We observe no staining with the V5 antibody in the ovaries of *yw* flies, which do not encode Ao-V5, although we observe staining with DAPI and the MxC antibody. Thus, the V5 staining observed in Fig. 2C and Figures S10-S13 is specific to Ao-V5.

**Supplementary Figure S15.** We observe no staining in the absence of primary antibodies in ovaries from Ao-V5-expressing flies (“V5” and “Mxc” refer to staining with corresponding secondary antibodies only). Thus, the staining observed in Fig. 2C and Figures S10-S13 does not result from artefactual staining with secondary antibodies.

**Supplementary Figure S16. Histone expression levels in unfertilized eggs from ∆*ao* or *ao^1^* females. (A)** We used RT-qPCR on 0-7 hour unfertilized eggs from 3-7 day old virgin ∆*ao* or isogenic *yw* females to assess levels of histone expression. We used RNA from 10 unfertilized eggs for each genotype. The median of the technical triplicate is shown. Gene expression has been normalized to *rp49.* We found no evidence for significantly elevated histone expression in ∆*ao* relative to wildtype (dashed line) except for a slightly increased histone H1 expression (consistent with Fig. 3A). **(B)** We used RT-qPCR on unfertilized eggs from *ao^1^* females relative to *yw* females. Expectedly, ao expression is not detectable in *ao^1^* unfertilized eggs. The expression levels for most histones are also not significantly deviant from wildtype (dashed line). However, histone H2B levels are significantly lower, just as they are in ovaries from *ao^1^* females (Fig. 3B) (data in Table S8).

**Supplementary Figure S17. Introducing a histone deficiency in a *∆ao* strain.** The endogenous *ao* and the histone loci are encoded on the *D. melanogaster* 2^nd^ chromosome near each other, with *ao* present on 32C and histone genes on 39DE cytological locations. To produce a fly homozygous for *∆ao* and carrying a heterozygous histone deficiency (Fig. 4A), we used recombination in female flies heterozygous for *∆ao* and the histone deficiency, and visual screening (*ao* is replaced by *dsRed* in *∆ao*) and PCR-mediated screening for loss of the histone locus to recover flies carrying *∆ao* and the histone deficiency on the same chromosome.

**Supplementary Figure S18. Constructing different histone gene copy number strains in D. melanogaster strains. (A)** Typically, *D. melanogaster* strains encode core, replication-coupled histones (H2A, H2B, H3B, H4) and the linker histone H1 in a multigene array, which is repeated 100 times at the 39DE cytological location on the 2^nd^ chromosome. Recent efforts have also introduced a 12xhistone transgene array at the 86F6 cytological locus on the 3^rd^ chromosome. By taking advantage of the presence or absence of each of these arrays, we can produce *D. melanogaster* strains encoding different histone gene copy numbers, ranging from 224 copies (homozygous for both endogenous histone locus alleles and 12xhistone arrays) to 24 copies (homozygous deletion of both endogenous histone locus alleles but presence of two 12xhistone arrays), and several intermediate configurations of 200, 124, and 100 copies. We used these histone gene configurations to investigate the relationship between histone gene copy number and *ao* expression (Fig. 3B).
