# Supplementary material for: The Drosophila maternal-effect gene abnormal oocyte (ao) does not repress histone gene expression
Source: bioRxiv. 2024 Sep 18:2024.09.17.613536. Preprint. [Version 1] doi: 10.1101/2024.09.17.613536 (PMC11429765; doi:10.1101/2024.09.17.613536)
Supplement: Supplement 2 [file media-2.pdf]

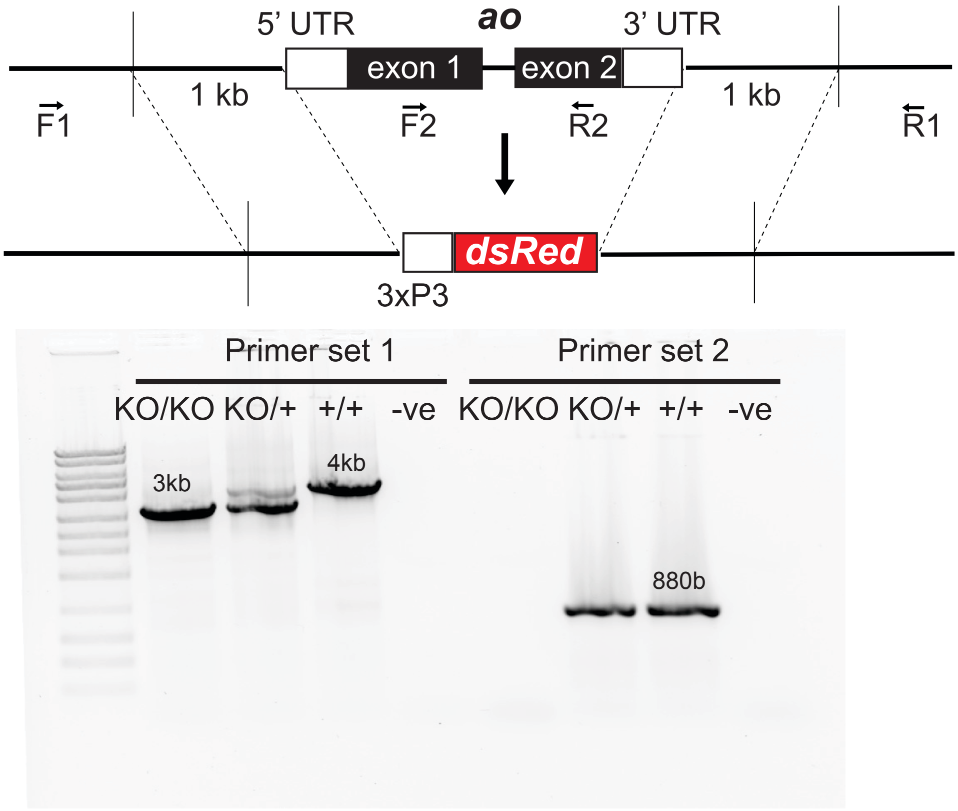
ao
5' UTR
3' UTR
exon 1
exon 2
1 kb
1 kb
F1
F2
R2
R1
dsRed
3xP3
Primer set 1
Primer set 2
KO/KO KO/+ +/+ -ve
KO/KO KO/+ +/+ -ve
3kb
4kb
880b

Figure S1

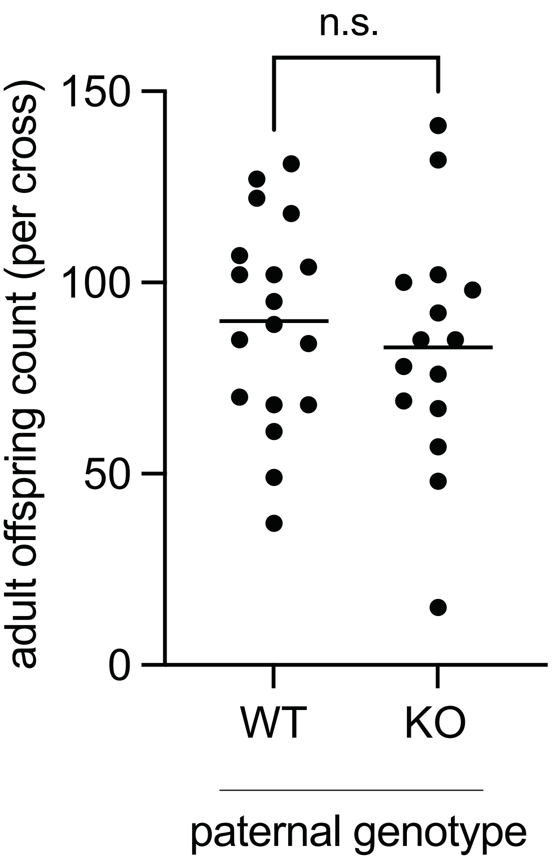
n.s.
150
100
50
0
adult offspring count (per cross)
WT
KO
paternal genotype

Figure S2

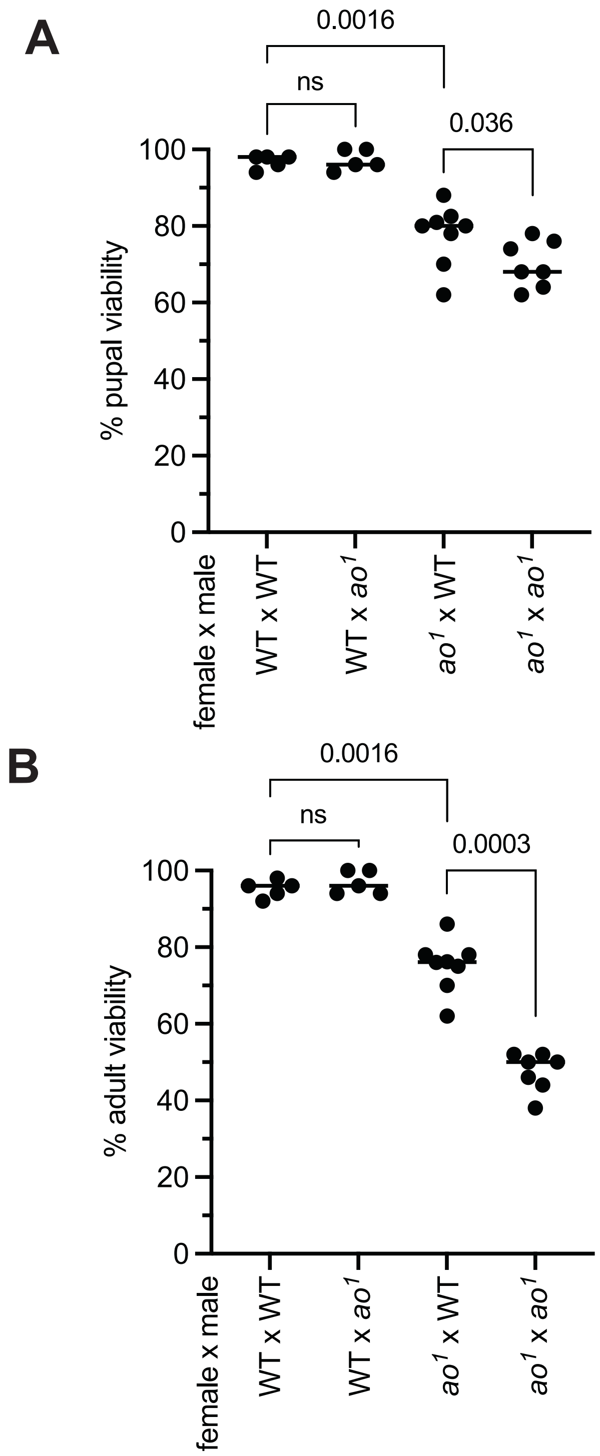
A
0.0016
ns
0.036
% pupal viability
100
80
60
40
20
0
female x male
WT x WT
WT x ao1
ao1 x WT
ao1 x ao1
B
0.0016
ns
0.0003
% adult viability
100
80
60
40
20
0
female x male
WT x WT
WT x ao1
ao1 x WT
ao1 x ao1

Figure S3

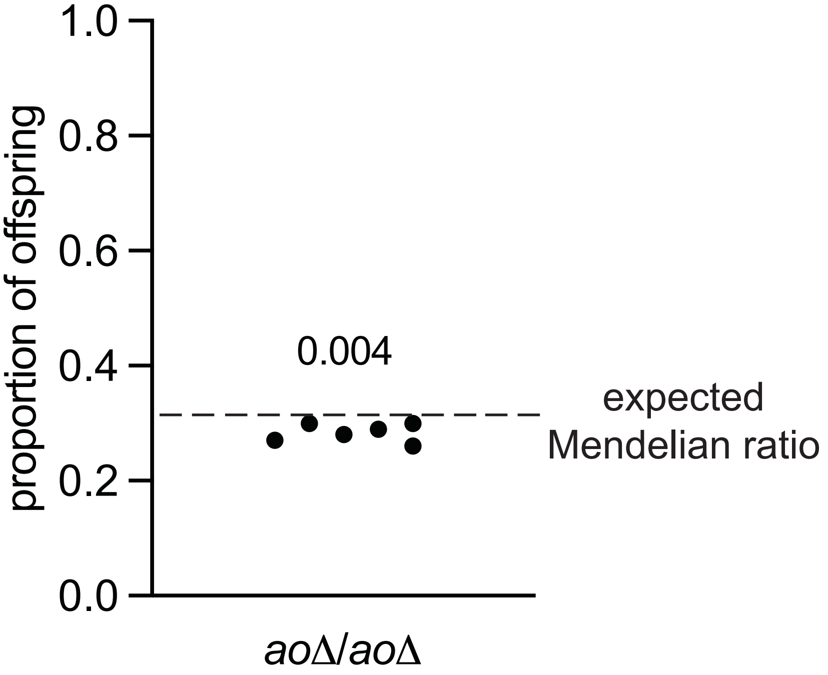
1.0
0.8
0.6
0.4
0.2
0.0
proportion of offspring
0.004
expected
Mendelian ratio
aoΔ/aoΔ

Figure S4

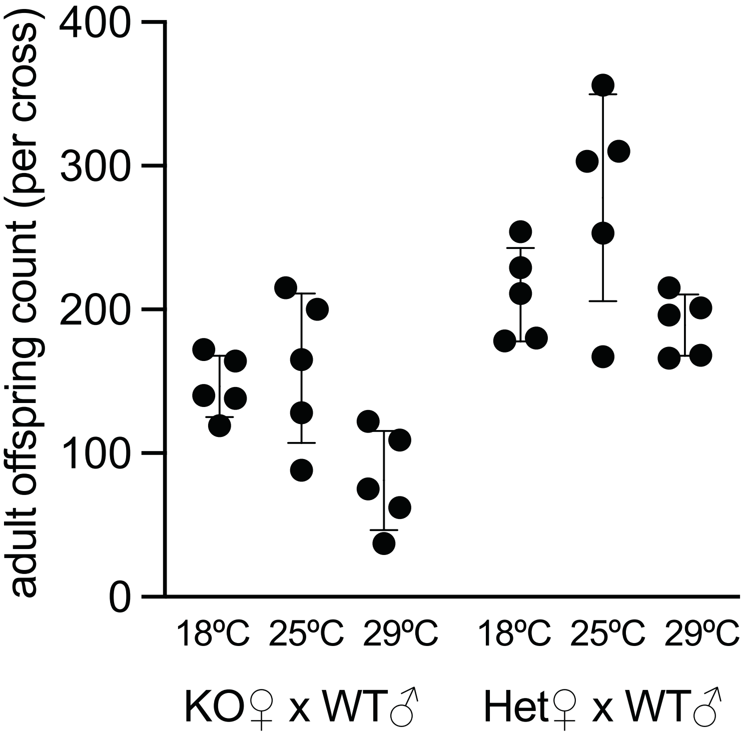
adult offspring count (per cross)
400
300
200
100
0
18ºC
25ºC
29ºC
18ºC
25ºC
29ºC
KO♀ x WT♂
Het♀ x WT♂

Figure S5

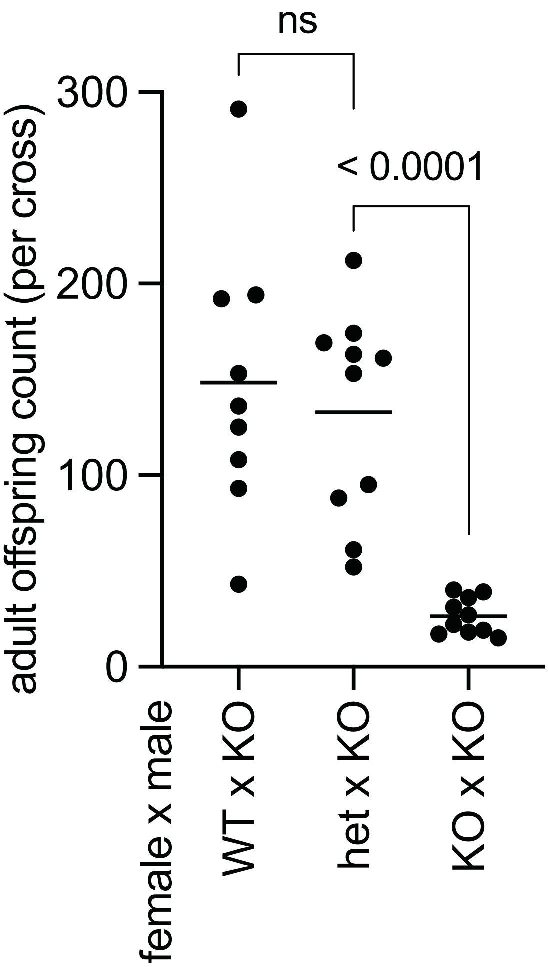
ns
< 0.0001
adult offspring count (per cross)
300
200
100
0
female x male
WT x KO
het x KO
KO x KO

Figure S6

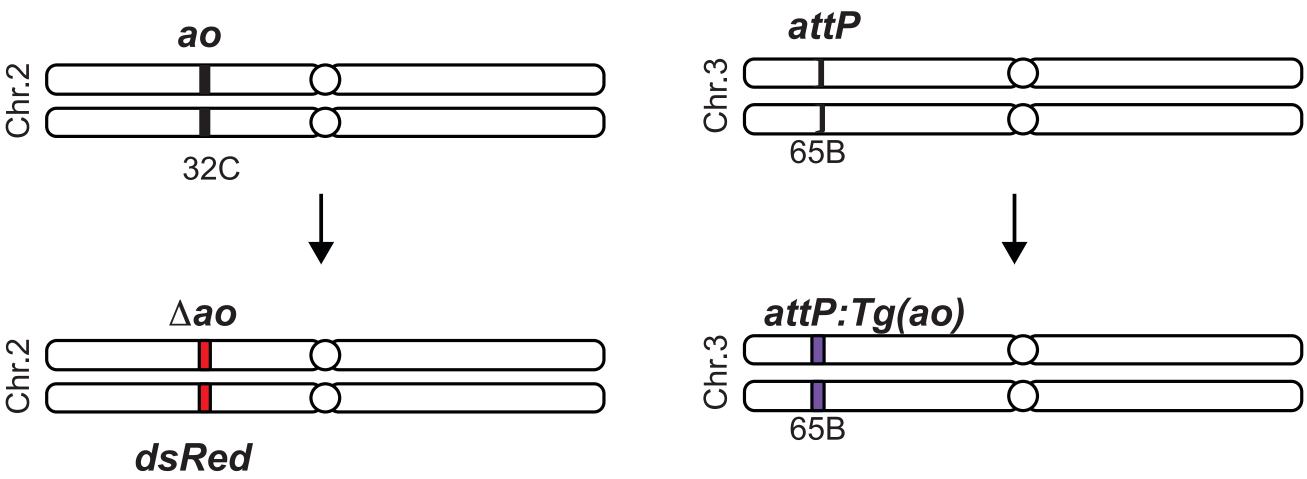
ao
Chr.2
32C
Δao
Chr.2
dsRed
attP
Chr.3
65B
attP:Tg(ao)
Chr.3
65B

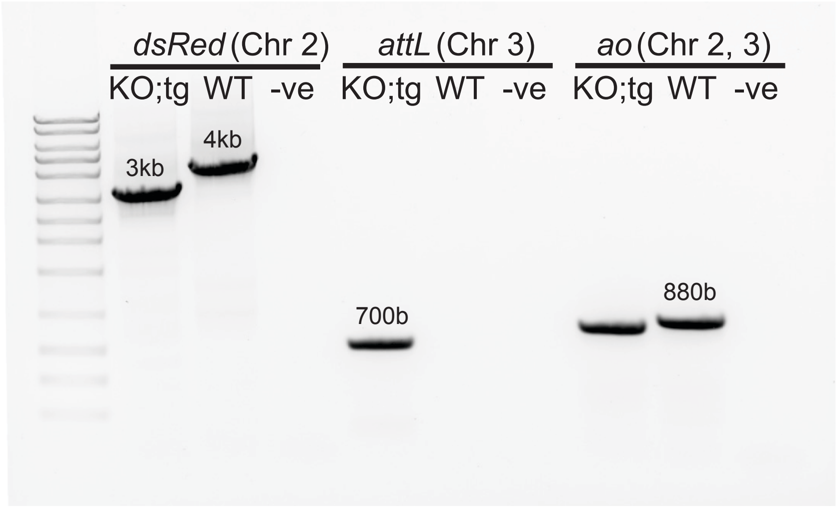
dsRed (Chr 2)
attL (Chr 3)
ao (Chr 2, 3)
KO;tg WT -ve
KO;tg WT -ve
KO;tg WT -ve
3kb
4kb
700b
880b

Figure S7

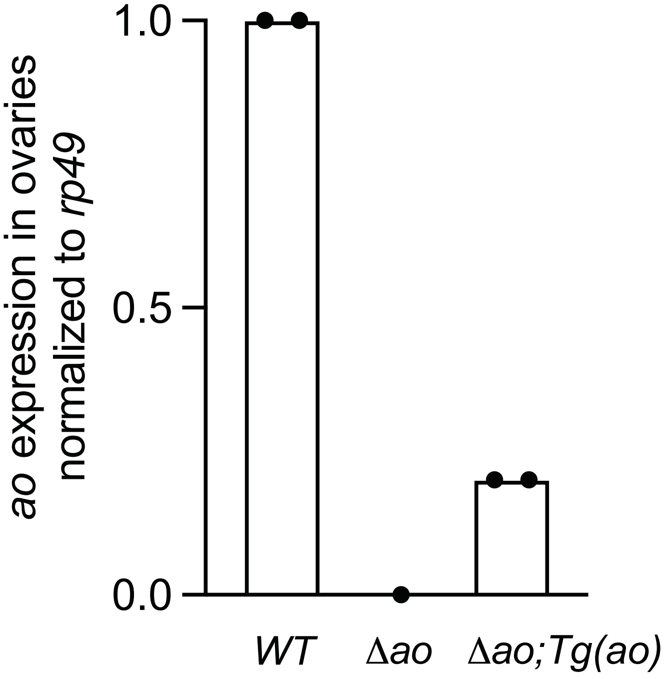

1.0
0.5
0.0
ao expression in ovaries normalized to *rp49*
WT
Δ*ao*
Δ*ao;Tg(ao)*

Figure S8

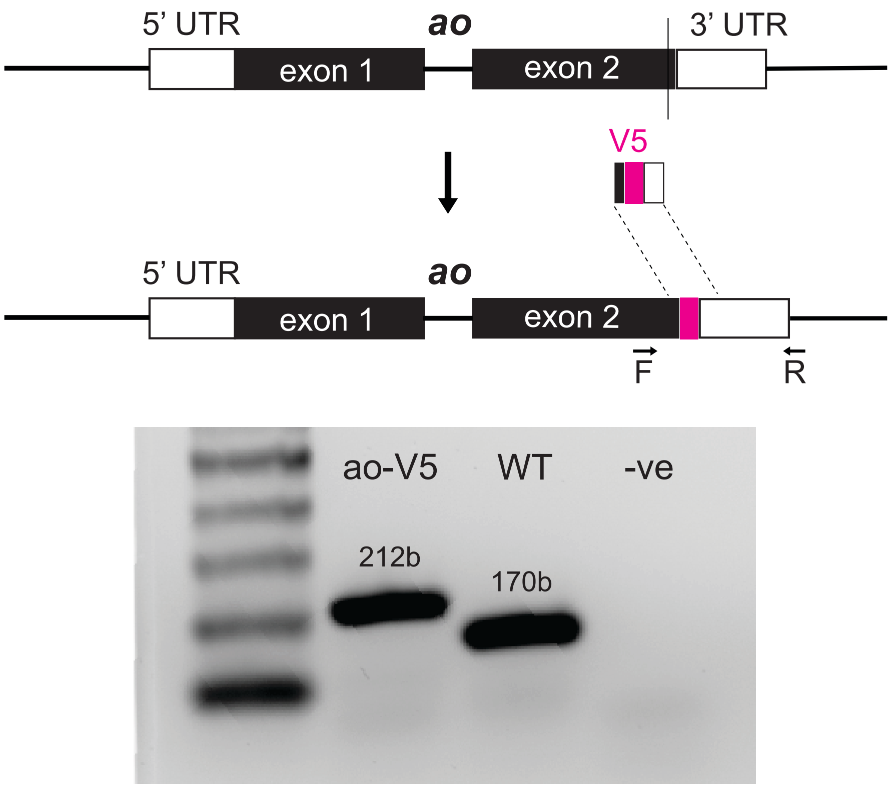
5' UTR
ao
3' UTR
exon 1
exon 2
V5
5' UTR
ao
exon 1
exon 2
F
R
ao-V5
WT
-ve
212b
170b

FIgure S9

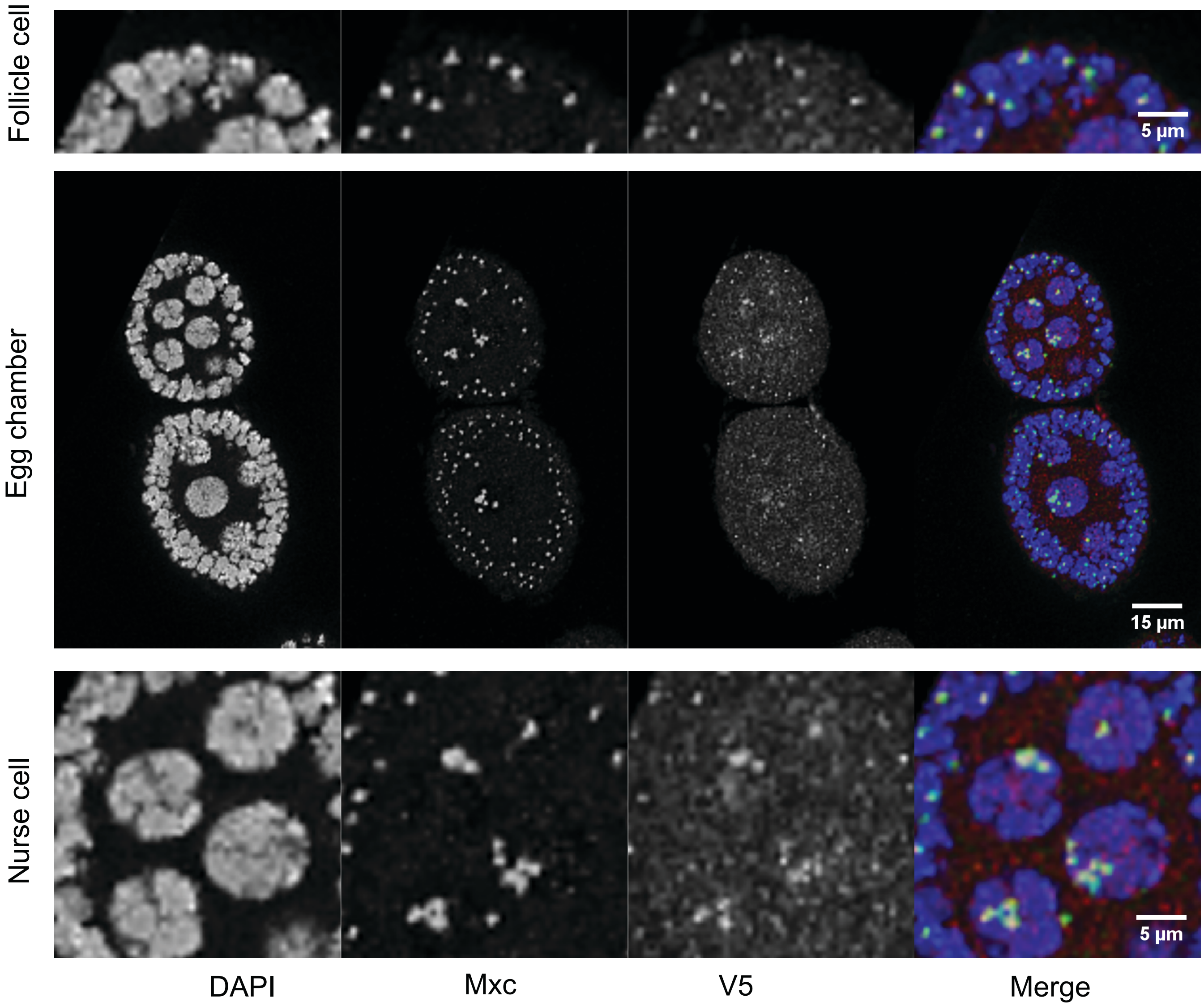
Follicle cell
Egg chamber
Nurse cell
5 µm
15 µm
5 µm
DAPI
Mxc
V5
Merge

Figure S11

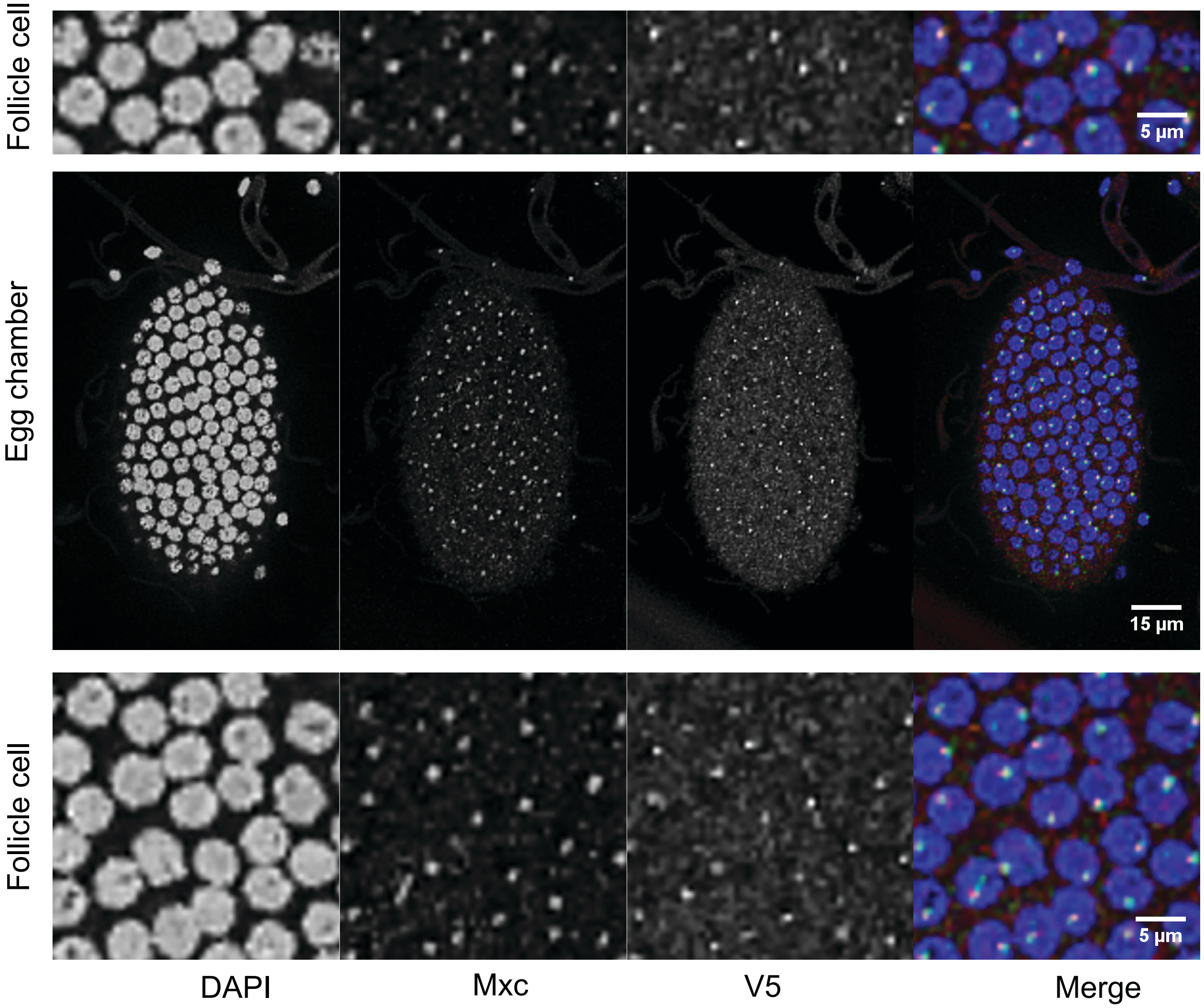

Follicle cell
Egg chamber
Follicle cell
5 µm
15 µm
5 µm
DAPI
Mxc
V5
Merge

Figure S10

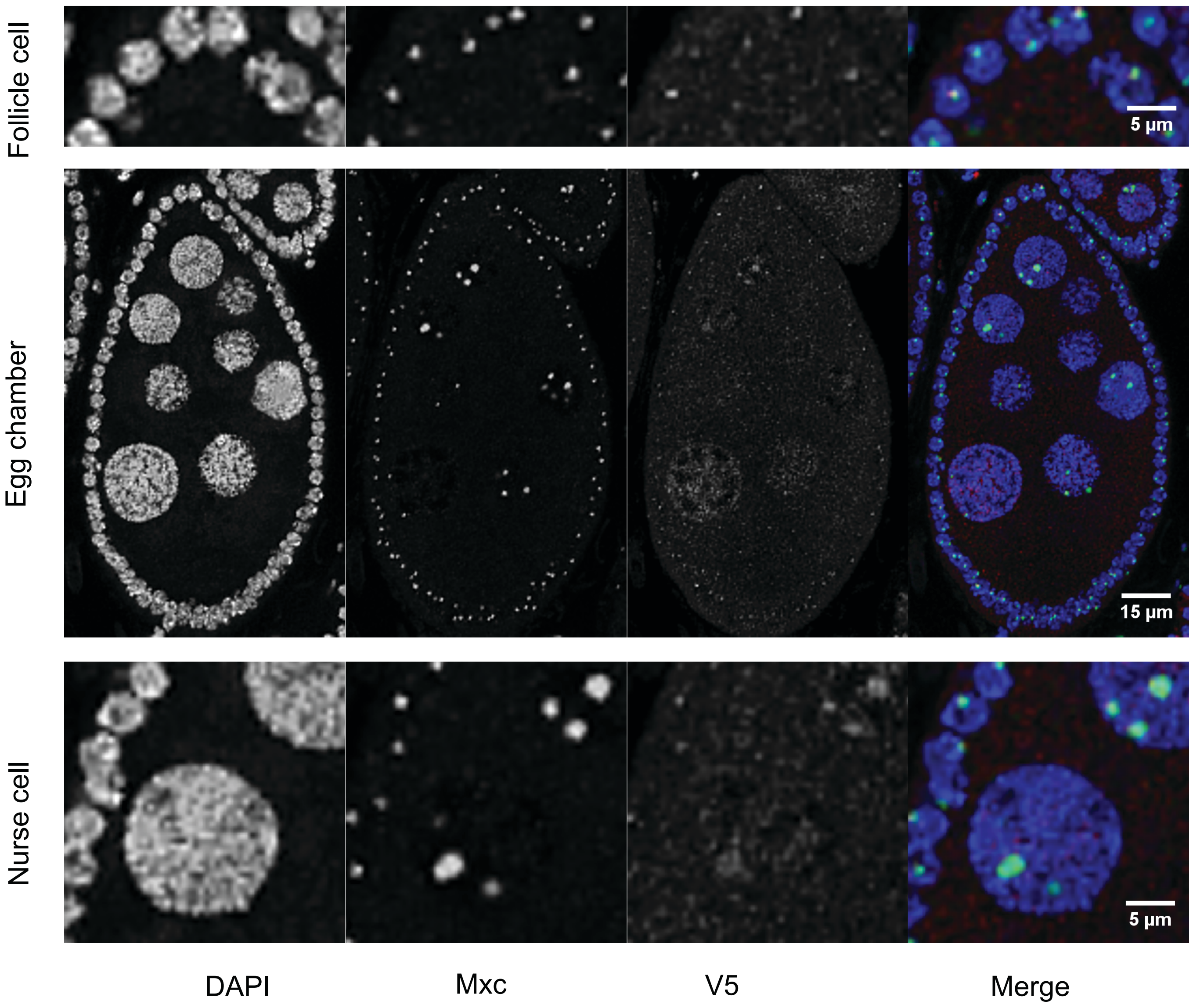
Follicle cell
Egg chamber
Nurse cell
5 µm
15 µm
5 µm
DAPI
Mxc
V5
Merge

Figure S12

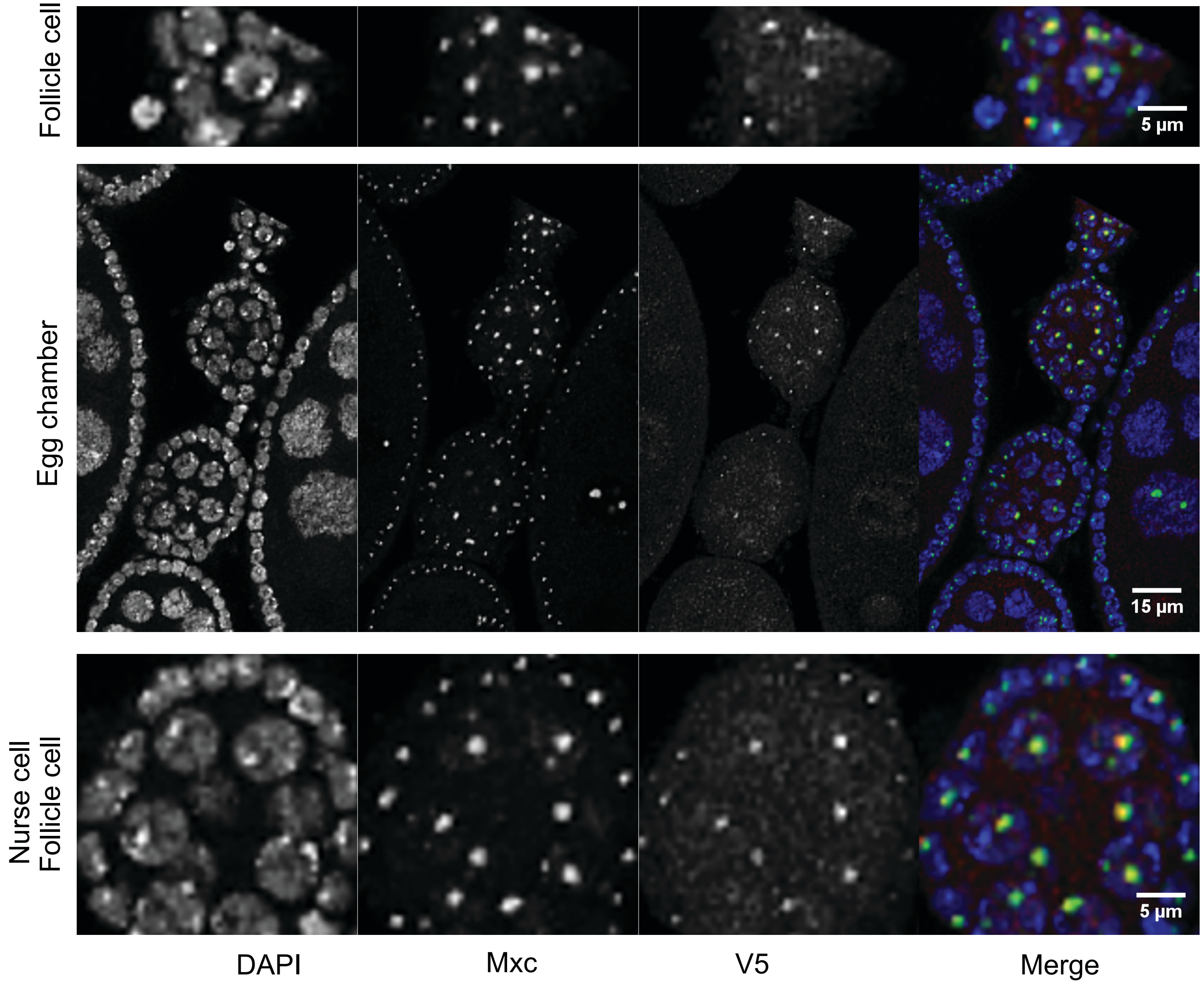
Follicle cell
Egg chamber
Nurse cell
Follicle cell
5 µm
15 µm
5 µm
DAPI
Mxc
V5
Merge

Figure S13

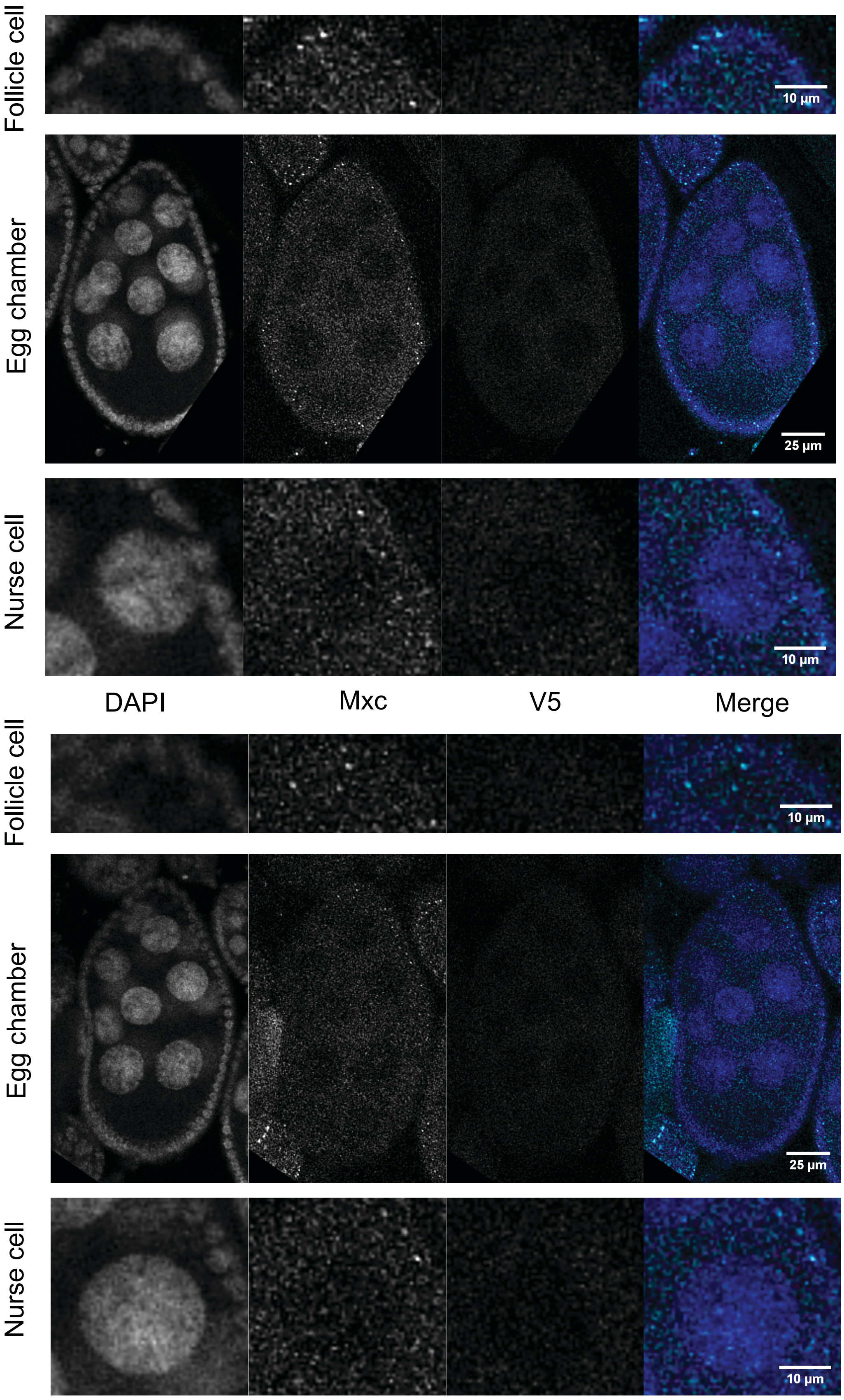

Follicle cell
10 µm
Egg chamber
25 µm
Nurse cell
10 µm
DAPI
Mxc
V5
Merge
Follicle cell
10 µm
Egg chamber
25 µm
Nurse cell
10 µm

Figure S14

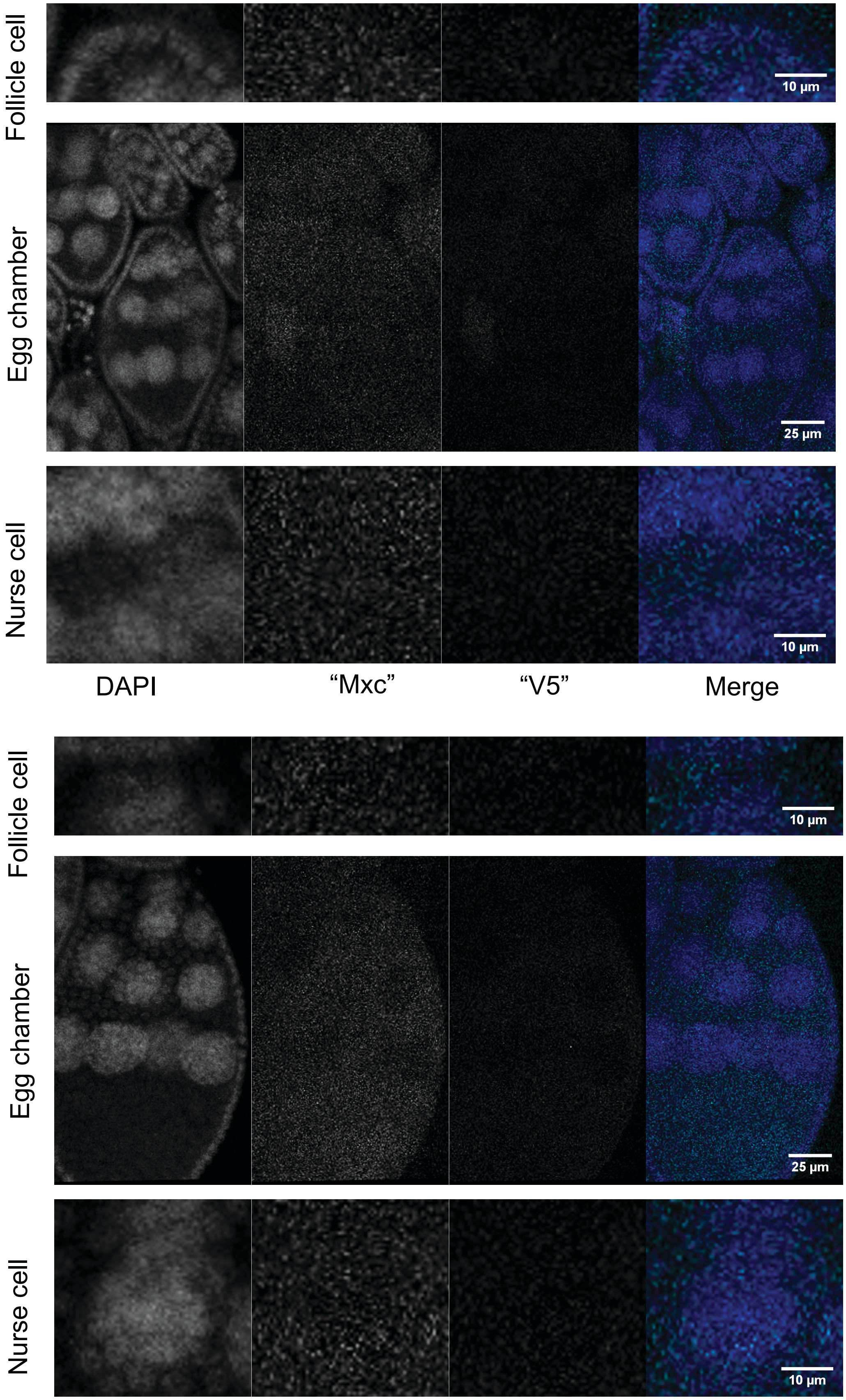
Follicle cell
Egg chamber
Nurse cell
10 µm
25 µm
10 µm
DAPI
“Mxc”
“V5”
Merge
Follicle cell
Egg chamber
Nurse cell
10 µm
25 µm
10 µm

Figure S15

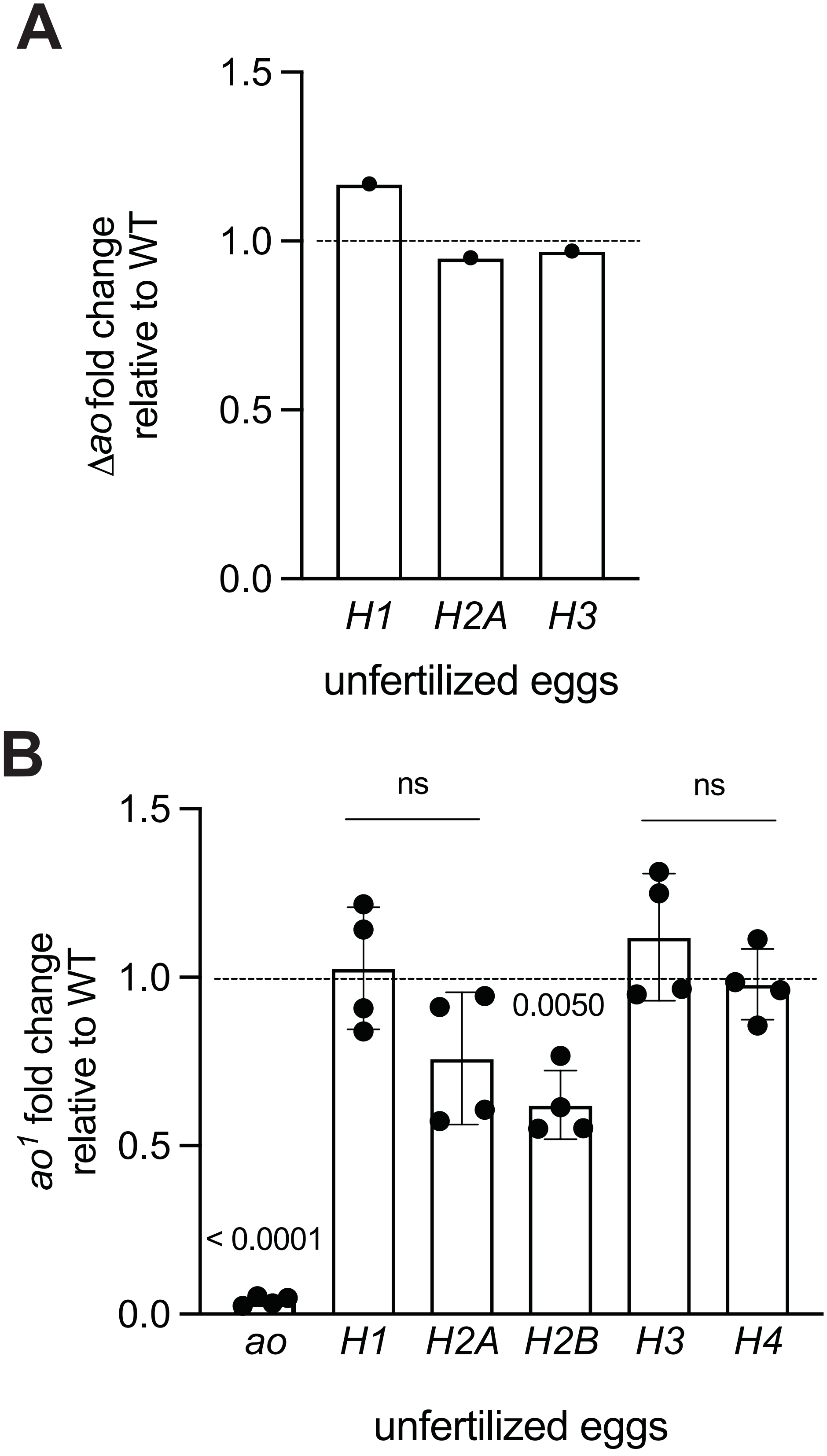
A
1.5
1.0
0.5
0.0
Δao fold change relative to WT
H1
H2A
H3
unfertilized eggs
B
ns
ns
1.5
1.0
0.5
0.0
0.0050
< 0.0001
ao1 fold change relative to WT
ao
H1
H2A
H2B
H3
H4
unfertilized eggs

FIgure S16

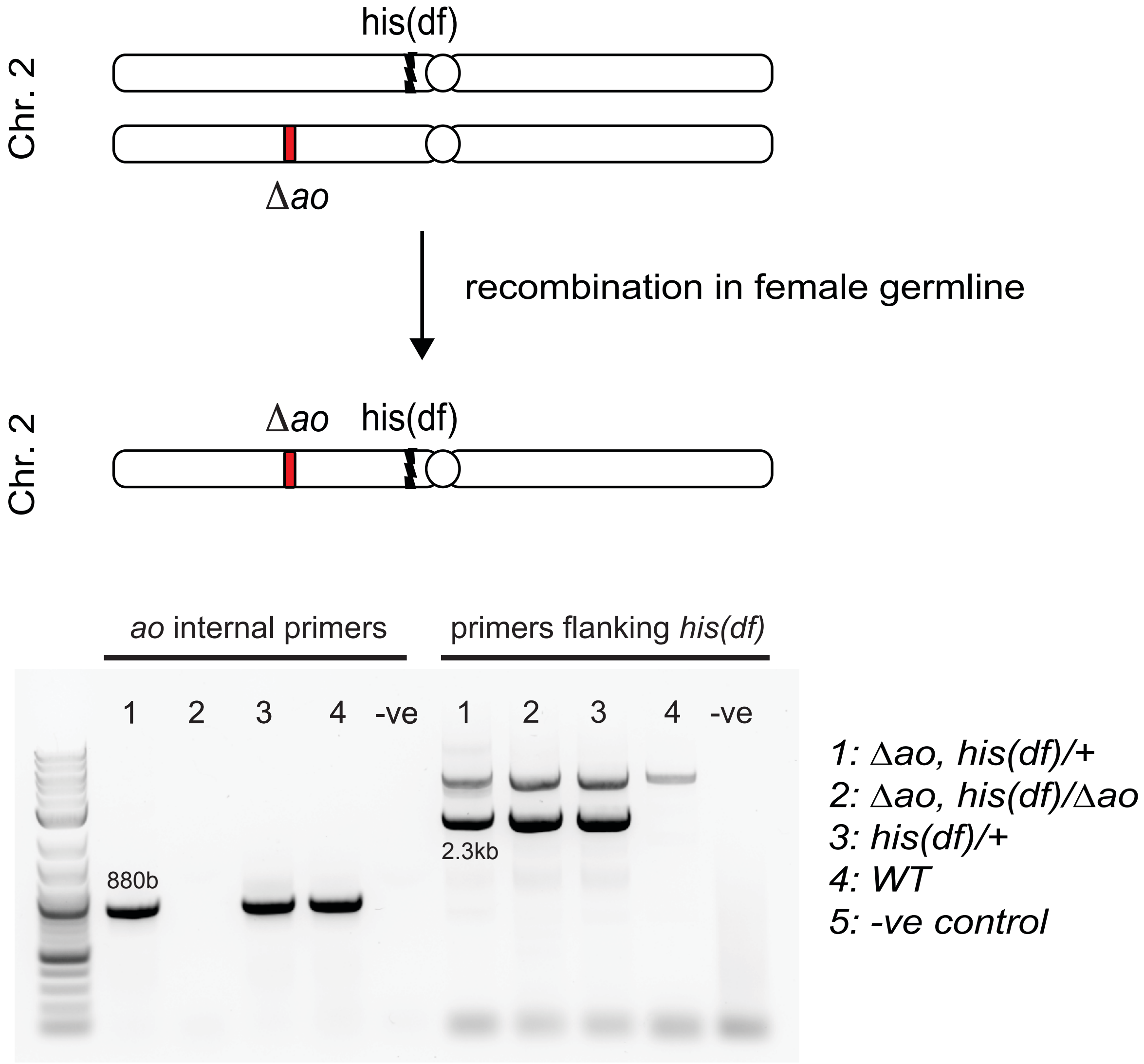
his(df)
Chr. 2
Δao
recombination in female germline
Δao his(df)
Chr. 2
ao internal primers
primers flanking his(df)
1 2 3 4 -ve 1 2 3 4 -ve
880b
2.3kb
1: Δao, his(df)/+
2: Δao, his(df)/Δao
3: his(df)/+
4: WT
5: -ve control

Figure S17

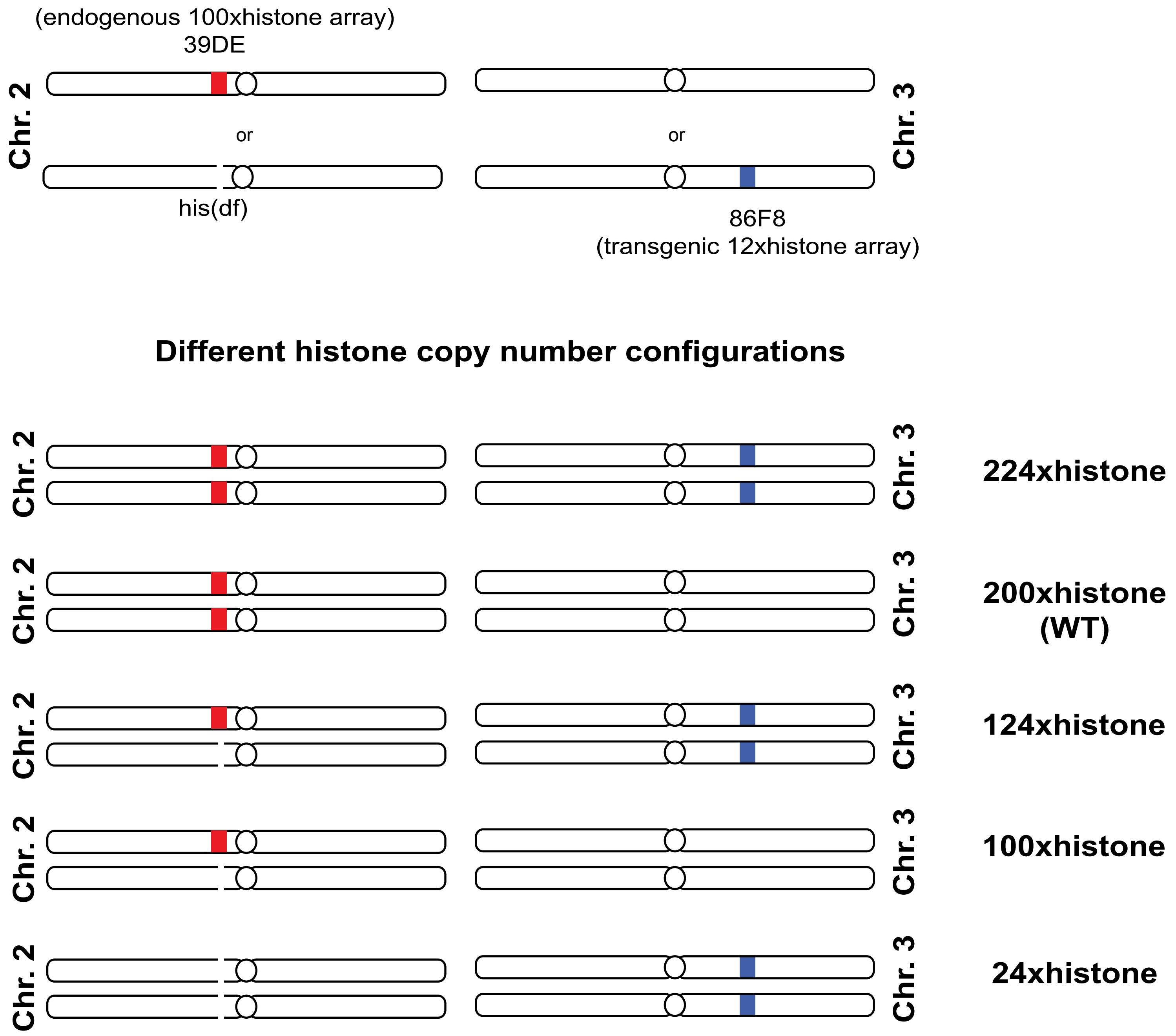
(endogenous 100xhistone array)
39DE
Chr. 2
or
his(df)
or
Chr. 3
86F8
(transgenic 12xhistone array)
Different histone copy number configurations
224xhistone
200xhistone (WT)
124xhistone
100xhistone
24xhistone

Figure S18
